# Filament structure and subcellular organization of the bacterial intermediate filament–like protein crescentin

**DOI:** 10.1073/pnas.2309984121

**Published:** 2024-02-07

**Authors:** Yue Liu, Fusinita van den Ent, Jan Löwe

**Affiliations:** ^a^Medical Research Council Laboratory of Molecular Biology, Structural Studies Division, Cambridge Biomedical Campus, Cambridge CB2 0QH, United Kingdom

**Keywords:** CreS, cell shape, filamentous proteins, bacterial cytoskeleton, cryo-EM

## Abstract

The protein crescentin is required for the crescent shape of the freshwater bacterium *Caulobacter crescentus* (*vibrioides*). Crescentin forms a filamentous structure on the inner, concave side of the curved cells. It shares features with eukaryotic intermediate filament (IF) proteins, including the formation of static filaments based on long and parallel coiled coils, the protein’s length, structural roles in cell and organelle shape determination and the presence of a coiled coil discontinuity called the “stutter.” Here, we have used electron cryomicroscopy (cryo-EM) to determine the structure of the full-length protein and its filament, exploiting a crescentin-specific nanobody. The filament is formed by two strands, related by twofold symmetry, that each consist of two dimers, resulting in an octameric assembly. Crescentin subunits form longitudinal contacts head-to-head and tail-to-tail, making the entire filament non-polar. Using in vivo site-directed cysteine cross-linking, we demonstrated that contacts observed in the in vitro filament structure exist in cells. Electron cryotomography (cryo-ET) of cells expressing crescentin showed filaments on the concave side of the curved cells, close to the inner membrane, where they form a band. When comparing with current models of IF proteins and their filaments, which are also built from parallel coiled coil dimers and lack overall polarity, it emerges that IF proteins form head-to-tail longitudinal contacts in contrast to crescentin and hence several inter-dimer contacts in IFs have no equivalents in crescentin filaments. Our work supports the idea that intermediate filament-like proteins achieve their shared polymerization and mechanical properties through a variety of filament architectures.

## Significance

Crescentin is a coiled coil protein that is required for the crescent cell shape of bacteria such as *Caulobacter crescentus*. Crescentin shares biochemical and cytoskeletal properties with intermediate filament (IF) proteins, which form the third major class of cytoskeletal proteins in eukaryotes. To better understand the relationship between crescentin and IF proteins, and the filaments they form, we have determined the three-dimensional structure of crescentin filaments by cryo-EM. This revealed the full-length structure of the parallel coiled coil dimer of crescentin and how dimers come together laterally and longitudinally, to form a non-polar, octameric filament. Differences in filament architecture highlight the versatility of intermediate filament-like proteins across the tree of life.

Fitness of bacteria in the environment is often dependent on their shape. It is therefore that most bacteria tightly control their shape, producing spheres (cocci), rods (bacilli), helices, filaments, and irregular, appended as well as crescent shapes (1). In many cases, shape is dependent on the stress-bearing cell wall. Hence, shape determination is dependent on the ability to regulate cell wall synthesis and remodeling during vegetative growth, as well as during cell division (2).

*Caulobacter crescentus* (*vibrioides*) is crescent shaped. The gram-negative freshwater bacterium has been studied extensively and not only because its cells undergo a developmental transition from swarmer to stalked cells. The larger stalked cells are able to attach to surfaces via the stalk, while the swarmer cells are flagellated and motile. Only stalked cells replicate DNA and divide. Their cell division is asymmetric and leads to one swarmer cell and one stalked cell, closing the cell cycle (3). Both cell types are crescent-shaped. It is thought that the crescent shape is advantageous for cell mobility in their aqueous environment, but it has recently been suggested that the crescent shape enables pili on the swarmer cells to attach to surfaces for biofilm formation (4).

Whatever the biological reason for its crescent shape, in *Caulobacter,* it is dependent on the function of the protein crescentin (CreS) (5). Immunofluorescence and fluorescent protein-tagging using a merodiploid strain (because CreS-GFP fusions were non-functional) showed crescentin to form a single filamentous structure on the inner (concave) surface of the cell crescent (5–7). The proximity of the crescentin structure to the cell membrane was lost during treatment with the MreB inhibitor A22 or the cell wall inhibitors mecillinam and phosphomycin (6). Based on the cellular localization, the cytoskeletal properties of crescentin and modeling it has been proposed that its mode of action to create cell shape is mechanical (6). The finding that MreB is involved (7) might alternatively indicate that crescentin filaments reduce cell wall synthesis on one side of the cell, leading to the crescent shape. In line with these ideas, it was found that the heterologous expression of crescentin in *E*scherichia* coli* leads to curved cells (6), suggesting a conserved or very direct mechanism of cell wall synthesis inhibition.

Crescentin levels in cells do not vary much during the cell cycle (7). Crescentin filaments in cells are not dynamic and free molecules are added to the filamentous cellular structure rapidly, indicating strong cooperativity of assembly (7, 8). Initial assembly starts with a cell-long thin structure that thickens as the cell progresses through the cell cycle. Re-arrangements of the crescentin structure occur during cell division, although it is not known how those are regulated or facilitated (7).

The discovery of crescentin as a regulator of cell shape attracted a lot of attention also because of a range of similarities of crescentin to eukaryotic intermediate filament (IF) proteins (5, 9). These similarities include domain organization, the basic building block for assembly, biochemical properties, filament polymerization and dynamics, mechanical properties, as well as cellular functions. These similarities have prompted the categorization of a group of bacterial proteins, including crescentin, as “IF-like” proteins (10). IFs are the third major class of intracellular filaments in eukaryotes, in addition to microfilaments (actin) and microtubules (tubulin). IFs can be categorized into five major families and include two types of cytoplasmic keratins, vimentin, and neurofilament, as well as nuclear lamins (11). IFs have a well-defined domain architecture, with a central α-helical “rod” domain driving the formation of parallel coiled coil dimers. The rod domain, which has been sub-divided into three coiled coil segments (coil 1A, 1B and 2) and two inter-connecting linkers (L1 and L12), is complemented primarily by disordered head and tail domains (in contrast, nuclear lamins and invertebrate cytoplasmic IFs have folded Ig-fold tail domains). Cytoplasmic IFs have rod domains of about 308 residues, nuclear lamins of about 350 residues. All IF sequences have a “stutter” in the third rod segment that deviates from the regular coiled coil heptad repeat pattern. Despite many years of research, there is still no complete structural description of any IF (12). They are known to form tetramers through inter-dimer interactions mediated by their N-terminal regions of the rod domain (“A11” inter-dimer contact). Filaments are thought to contain tetramers as building blocks, at least for the cytoplasmic IFs, and three other types of inter-dimer contacts (“A22”, “A12”, and “ACN”) then lead to higher-order structures such as the prototypical 10 nm-thick mature fibers of many IF proteins (13).

Crescentin contains the heptad repeats needed to form an extended coiled coil dimer, and also a stutter in the C-terminal half. The rod domain of crescentin is longer than IF proteins, and it was demonstrated early on that crescentin forms very stable filaments in vitro (5). Filament formation in vitro and in vivo showed that the domain organization of crescentin is important for its polymerization and cell shape maintenance functions (9). Removal of the stutter also interfered with crescentin’s ability to curve cells (9). Membrane attachment was shown to depend on the first 27 amino acids (6, 9).

Given the enigmatic relationship between crescentin and IFs, our lack of understanding how crescentin filaments form in cells and how they control cell shape, we set out to understand the crescentin filament structure. We employed electron cryomicroscopy (cryo-EM) on crescentin filaments bound by a megabody (14) to determine their structure, which revealed an “octameric” twisted and double-stranded filament without polarity. We used in vivo site-specific cysteine cross-linking to demonstrate that many, if not all, features of the in vitro filament structure exist in crescentin filaments in cells. Electron cryotomography (cryo-ET) of *Caulobacter* cells expressing crescentin showed the filaments on the inner, concave side of cells, close to the inner membrane where they form a single, wide band. In addition to similarities such as octameric protofilaments and the lack of overall polarity and, detailed comparison with current models of IF proteins revealed also significant differences between their filament architectures.

## Results

### Development of a Megabody for CreS Structure Determination.

In line with previously reported assembly properties of crescentin (CreS) and of many eukaryotic IF proteins (5, 9, 13, 15–17), polymerization of purified, un-tagged *Caulobacter crescentus* (*vibrioides*) CreS upon a decrease of pH in vitro led to CreS aggregates, bundles, or irregular filaments that exhibited structural polymorphism. In some cases, cryo-EM micrographs of wild-type (wt) CreS, namely CreS_wt,_ polymerized at pH 7.0 showed unbundled filaments with a width of ~9 nm (*SI Appendix*, Fig. S1*A*). Two-dimensional (2D) class averages revealed two intertwined strands with a regular spacing of ~57 nm (distance between helical cross-overs) (*SI Appendix*, Fig. S1*B*). Nevertheless, the smooth appearance of these filaments prevented determination of the register of individual subunits along the filament axis.

**Fig. 1.**
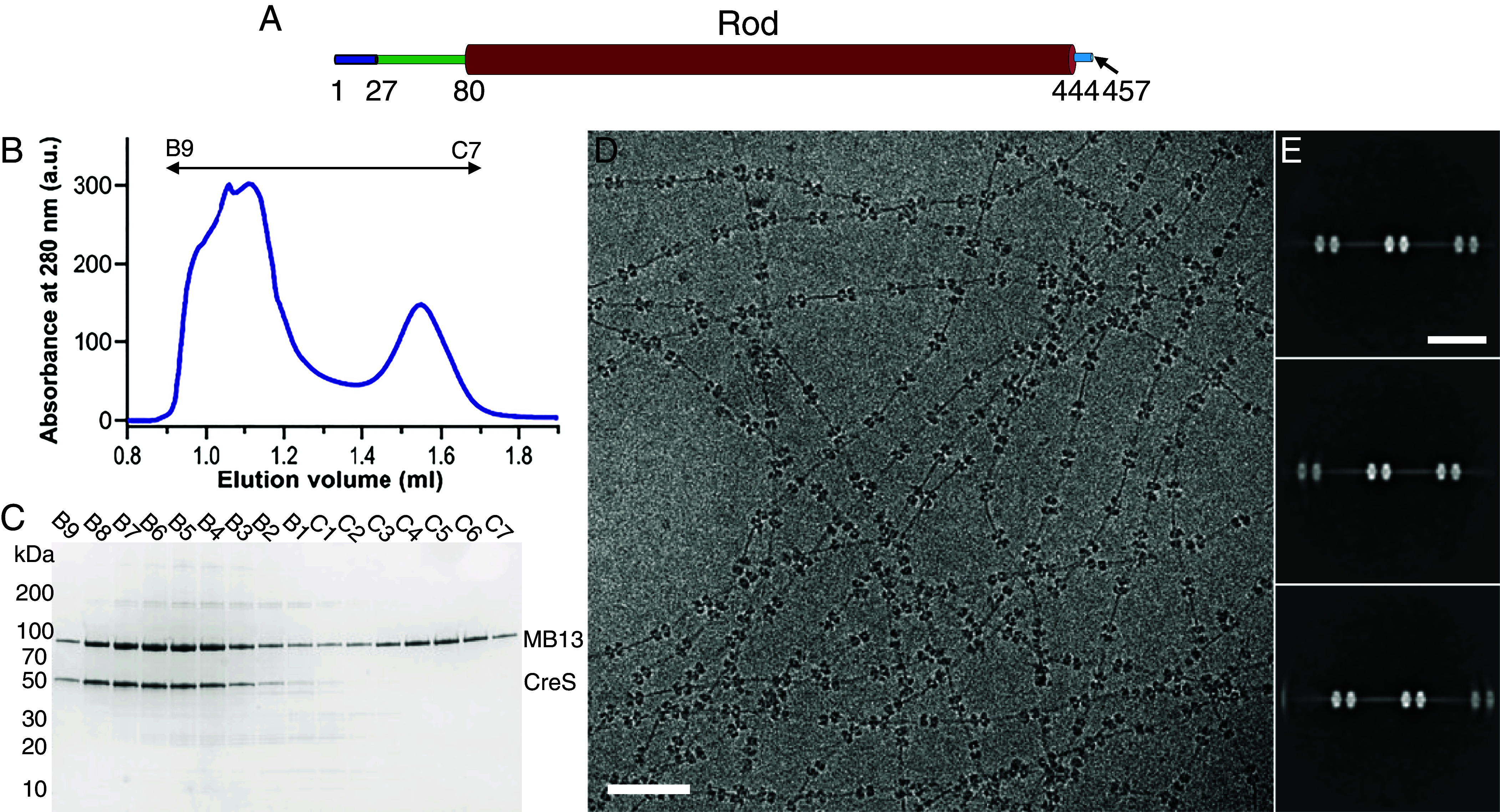
Production and characterization of a CreS-specific megabody (MB) for structure determination of CreS filaments. (*A*) Domain organization of full-length CreS. The rod domain was defined based on coiled coil prediction (18) and structure prediction using AlphaFold 2 (19) (*SI Appendix*, Fig. S1*C*). (*B*) Size exclusion chromatography (SEC) profile of CreS_sat_ [a mutant version that has a three amino acid insertion (Ser, Ala, and Thr; SAT) before position 406], preincubated with MB13 at 1:4 molar ratio at pH 8. See *SI Appendix*, Fig. S1 *D* and *E* for the SEC profile of CreS_wt_ mixed with MB13. (*C*) SDS-PAGE analysis of SEC fractions. Fractions B9 to B5 were pooled and used for EM studies. (*D*) A typical cryo-EM image showing single CreS filaments in complex with MB13 that are formed at pH 6.5. (*E*) Reference-free 2D classification of these filaments reveals a regular spacing of ~57 nm between neighboring “nodes” along the filament. The nodes are caused by MB binding.

To facilitate structure determination of CreS (see *SI Appendix*, Table S2 for protein sequences used), we raised nanobodies (NBs) against purified CreS_wt_ and selected NBs that bind to CreS_wt_ polymers formed at low pH conditions (*Materials and Methods*). Coiled coil prediction (18) and three-dimensional (3D) structure prediction by AlphaFold 2 (AF2) (19) (*SI Appendix*, Fig. S1*C*) indicated that CreS contains a central, coiled coil “rod” domain (residues 80–444), flanked by an N-terminal segment (residues 1–79, although somewhat shorter in AF2 predictions, *SI Appendix*, Fig. S1*C*) and a C-terminal segment (residues 445–457) (Fig. 1*A*). Given the elongated nature of the CreS coiled coil dimer, we engineered an NB derivative, megabody (MB), to act as a bulky and rigid marker bound to CreS. For each NB investigated, a MB was generated by grafting the NB onto a circular permutant of the scaffold protein *E. coli* YgjK, as previously described (14). Among the MBs, megabody 13 (MB13), a derivative of nanobody 13 (NB13) binds to both CreS_wt_ and CreS_sat_ at pH 8. CreS_sat_ contains a stretch of three amino acids (Ser, Ala, and Thr; SAT), inserted before residue 406, to remove the stutter that locally disrupts the continuity of the heptad-repeat coiled coil (9, 20) (Fig. 1 *B* and *C* and *SI Appendix*, Fig. S1 *D*–*F*). Cryo-EM analysis revealed that for both versions of CreS, purified CreS-MB13 complex at pH 8 polymerizes into regular, unbundled filaments upon lowering the pH to 6.5 in the presence of detergents, such as CHAPS (Fig. 1*D*). These filaments showed a regular spacing of ~57 nm between neighboring “nodes,” where a node is a segment of the CreS filament decorated with multiple MB13 molecules (Fig. 1*E* and *SI Appendix*, Fig. S3*A*).

**Fig. 2.**
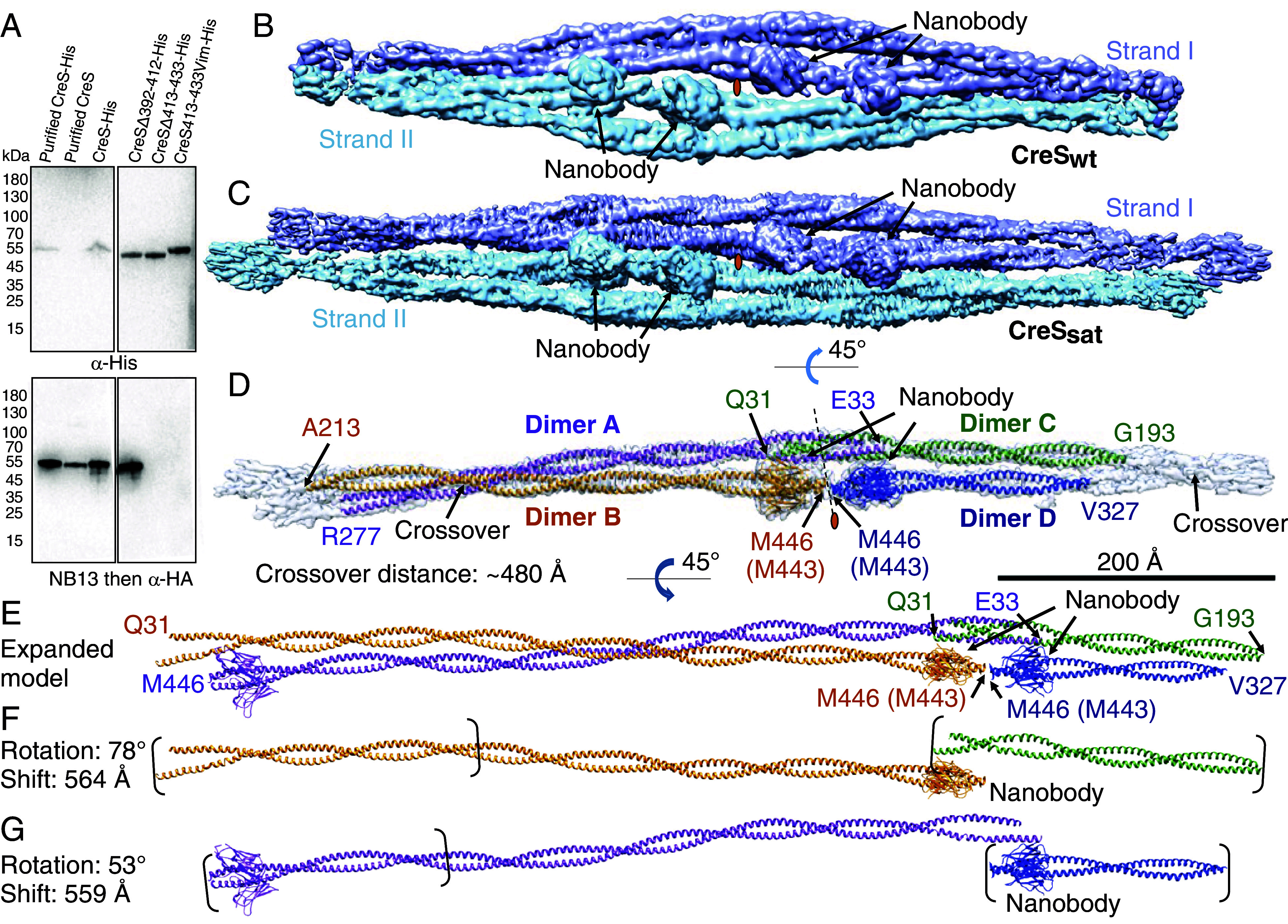
Architecture and assembly of CreS filaments. (*A*) Mapping of the CreS-specific nanobody (NB13) binding region on CreS in vitro. All samples except for purified proteins as indicated were cell lysates. The samples were immunoblotted with α-His for analysis of protein expression and with NB13 followed by α-HA for analysis of NB13 binding to CreS. Cryo-EM shows that CreS_wt_ (*B*) and CreS_sat_ (*C*) filaments share a common architecture formed by two strands (shown in cyan and blue) that twist around each other. (*D*) Each strand, with roughly the length of an asymmetric unit shown, consists of two CreS parallel coiled coil dimers that are held together via inter-dimer interactions. (*E*) The expanded atomic model reveals the structure of nearly complete CreS molecules, mostly missing N-terminal residues before Q31 that are presumably disordered. (*F* and *G*) Transformation of individual CreS dimers along the filament axis in a roughly linear fashion produces the observed single-stranded filamentous assembly. Panels *B*–*G* are drawn to scale (black scale bar: 200 nm). The positions of pseudo twofold symmetry axes are indicated by an orange oval. All CreS atomic models shown are of CreS_sat_ throughout all figure panels unless otherwise stated. In these cases, residues are numbered based on CreS_sat,_ whereas residue numbers in parentheses are according to CreS_wt._

For cryo-EM structure determination of CreS, we treated nodes as single particles, using a box size of ~420 Å (henceforth referred to as “small box”), to obtain well-resolved reconstructions of CreS bound with NB13 molecules (*Materials and Methods* and *SI Appendix*, Table S3 and Fig. S2). We next extended the box size to ~960 Å (referred to as “large box”) for the visualization of a more complete CreS structure, which included regions that are distant from the NB13 binding site, which was mapped to a segment near the C terminus of CreS (*Materials and Methods*, Fig. 2*A*, and *SI Appendix*, Table S3 and Fig. S2). The best small box and large box reconstructions were of CreS_sat_ complexed with NB13, determined at nominal resolutions of 3.34 Å (3.79 Å against the fitted atomic model) and 4.11 Å (4.75 Å against the fitted atomic model), respectively (*SI Appendix*, Fig. S3 *B*–*E* and Table S3). The register of individual amino acids on CreS_sat_ was assigned based on the binding site of NB13 on CreS and the observed patterns of well-resolved side chain densities (*Materials and Methods* and *SI Appendix*, Fig. S3*B*). The resulting atomic models of CreS_sat_ and NB13 allowed for interpretation of CreS_wt_ maps, determined at lower resolutions (4.36 Å to 5.78 Å) (*SI Appendix*, Fig. S4 *A* and *B* and Table S3). The atomic structure of CreS_wt_ resembles that of CreS_sat_, with only minor differences in regions near and following the stutter.

### Architecture of CreS Filaments.

In the presence of MB13, CreS_wt_, and CreS_sat_ similarly assemble into a “supertwist”-like filament, where two intertwined strands, I and II, are related by a two-fold axis perpendicular to the filament axis (Fig. 2 *B* and *C*). When looking at the map down this twofold axis, each strand consists of four partial CreS dimers (dimers A, B, C, and D for strand I, and symmetry-related dimers A’, B’, C’, and D’ for strand II) within a viewing window of ~960-Å wide along the filament. Each dimer is a parallel, polar coiled coil of CreS molecules. Strands I and II are held together via interactions between CreS dimers. Taking strand I as an example, inter-dimer interactions are found between a pair of dimers near their N termini (A-C pair) or their C termini (B-D pair) in the longitudinal direction (i.e., along the filament axis) (Fig. 2*D*). Hence, the two-stranded CreS filament presented here lacks polarity, as is the case for the individual strands on their own. This is reminiscent of eukaryotic intermediate filaments (IFs), which are also known to be non-polar (21–24). Furthermore, each pair of longitudinally associated dimers are structurally similar to each other, especially at the regions close to the inter-dimer interface near the N termini or C termini. Thus, every pair of longitudinally associated dimers are related by a local pseudo twofold axis perpendicular to the inter-strand twofold axis mentioned above (Fig. 2*D*). Each pair of laterally interacting dimers (A-B pair or C-D pair) alternate at a cross-over due to them twisting around each other, giving rise to a cross-over distance of ~480 Å along the strand (Fig. 2*D*).

Assembly of a nearly complete CreS dimer (residues 31–446 for CreS_sat_ or 31–443 for CreS_wt_) was possible through symmetry expansion, based on the observed partial dimers A and B, resulting in an atomic model of the strand (Fig. 2*E*). For this, the partial dimer C was superimposed with the equivalent part of the expanded dimer B upon a shift of 564 Å and a rotation of 78° (Fig. 2*F*). Likewise, the partial dimer D was superimposed with the equivalent region of the expanded dimer A upon a shift of 559 Å and a rotation of 53° (Fig. 2*G*). The model revealed that every strand has a regular spacing of ~560 Å, which corresponds to the length of a CreS molecule in a coiled coil. As a result, these transformations enable each strand and the two-stranded filament to propagate in a roughly linear fashion.

### Assembly of CreS Filaments.

**Fig. 3.**
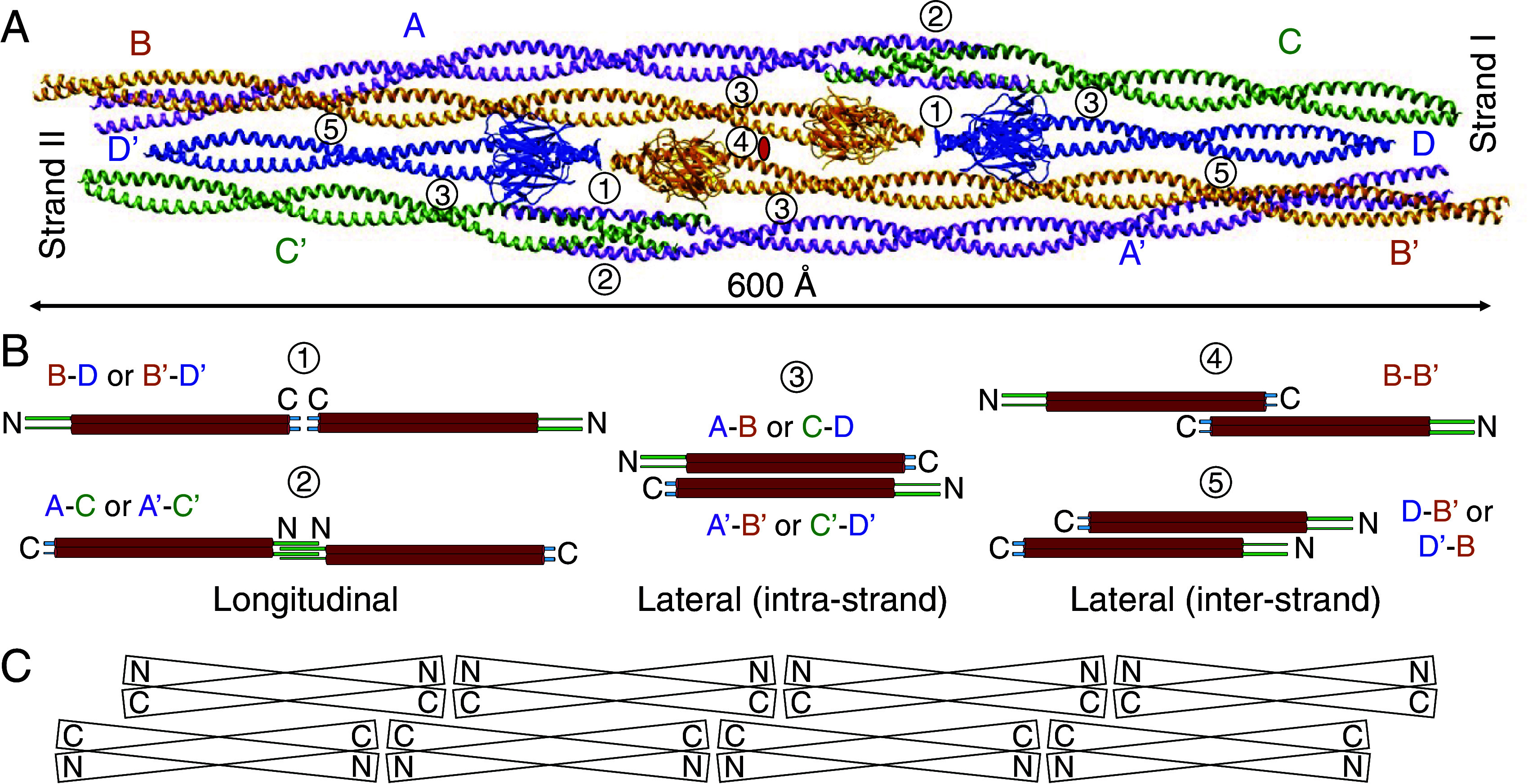
Contacts between CreS coiled coil dimers. (*A*) CreS coiled coil dimers assemble into the two-stranded filament structure via a multitude of longitudinal and lateral inter-dimer interactions that can be classified into five types. (*B*) Schematic representation of the five types of observed dimer–dimer interactions. (*C*) Schematic representation of the octameric assembly of crescentin as depicted in panel *A*.

A detailed structural analysis revealed five different types of dimer–dimer interactions that result in the observed CreS filament (Fig. 3*A*). Within each strand, there are both lateral and longitudinal interactions between CreS dimers. Interaction types 1 and 2 involve longitudinal inter-dimer interactions near the C termini (pairs B-D and B’-D’, residues ~443–457) and near the N termini (pairs A-C and A’-C’, residues ~31–83), respectively (Fig. 3*B* and *SI Appendix*, Fig. S4*C*). For inter-dimer interaction type 1, although residues 444–457 are disordered in our cryo-EM maps, residues 443 from different monomers not belonging to the same dimer are in close proximity to each other at the C termini. Type 3 represents lateral inter-dimer interactions (pairs A-B, C-D, A’-B’, C’-D’) (Fig. 3*B*). Dimer–dimer interactions between the two strands are primarily along the lateral direction, including type 4 (pair B-B’, antiparallel) and type 5 (pairs B-D’ and B’-D, parallel) (Fig. 3*B*). Whereas relatively short stretches of amino acids are responsible for longitudinal interaction types, all lateral interaction types cover long inter-dimer binding interfaces (Fig. 3*C*).

**Fig. 4.**
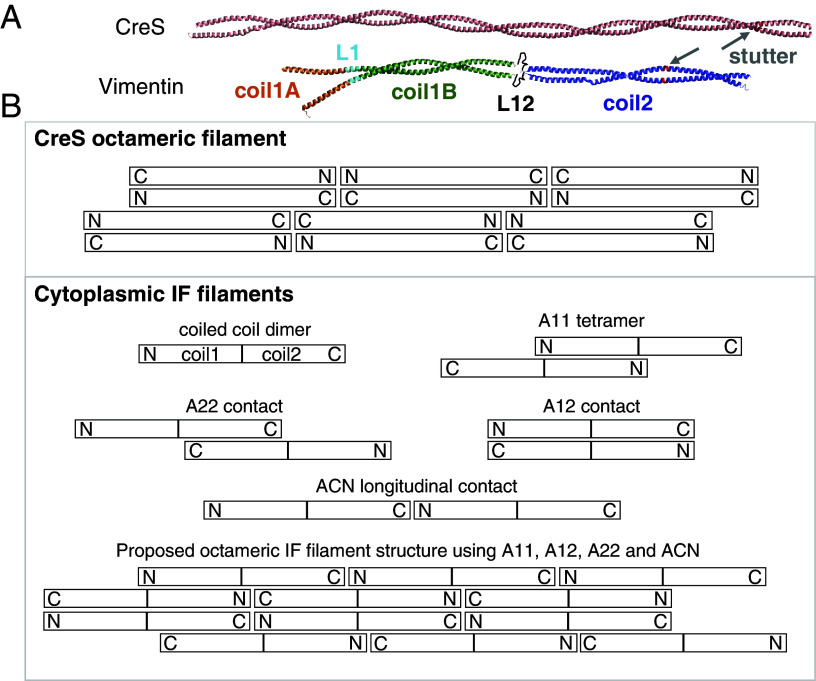
Structural comparison of CreS with eukaryotic IF proteins. (*A*) To-scale comparison of the atomic model of a CreS dimer (*Upper*) with the composite atomic model of a vimentin dimer (*Lower*). The IF structure shown is a hybrid model assembled from crystal structures of vimentin fragments (PDB entries 1GK4, 3S4R, 3TRT, 3UF1, and 1GK6), similar to what was reported (22). (*B*) Comparison of crescentin filaments (similar to Fig. 3*C*, but without the cross-overs shown for clarity) with current models of cytoplasmic IF protein filaments (lower box). IF proteins also form parallel and extended coiled coil dimers. Many IF proteins have been shown to form staggered and antiparallel “A11” tetramers, in which two dimers come together via their coil1 rod segments. IF polymers have also been shown, mostly by cross-linking studies, to contain staggered A22 and flush A12 contacts. End-to-end contacts are believed to be via N- and C-terminal ends coming together, head-to-tail ACN contacts. There is little experimental evidence for ACN contacts in cytoplasmic IFs, but it is difficult to build gap-free models using the other contacts, without ACN contacts. The current model of an IF “octameric” protofilament is shown at the bottom (12). The most striking difference to the structure of crescentin filaments identified here is that all longitudinal contacts are head-to-tail (N–C) in IFs (ACN) and not N–N and C–C head-to-head and head-to-tail, as in crescentin. Both filaments lack overall polarity and one inter-dimer contact is similar, A12 and interaction type 3 (Fig. 3), whereas the others are different. For example, there is no parallel inter-dimer contact in the IF model, as in crescentin’s interaction type 5 (Fig. 3). Furthermore, A11 and A22 in IFs are more substantial than the somewhat related contact types 2 (N–N) and 4 (C–C overlap) in crescentin, respectively (Fig. 3). While somewhat related in overall architecture, the way the dimers come together to form filaments can be described as significantly different.

As mentioned in the introductory paragraphs, the central rod domains of eukaryotic IF proteins are generally ~308 amino acids long, with the exceptions of nuclear lamins and invertebrate IF proteins (which are ~350 amino acids long). It has been proposed to be divided into three coiled coil segments with conserved lengths, 1A, 1B, and 2 (13), which are inter-connected by short linkers L1 and L12 (Fig. 4*A*). In contrast, CreS possesses a longer coiled coil rod domain (~364 amino acids), which does not show equivalent linkers that disrupt the overall α-helical structure, based on our cryo-EM maps (Fig. 4*A*). Both N-terminal and C-terminal segments of CreS are relatively short and do not contain complex domains as seen in many eukaryotic IF proteins (25). Furthermore, structural comparisons of CreS filamentous assemblies with those of eukaryotic IF proteins are hindered by a lack of atomic-level structural information concerning the assembly of eukaryotic IFs, despite decades of research (22, 26–29). Nevertheless, it has been established that the coiled coil dimer of IF proteins functions as the basic subunit for IF assembly (13). Four types of inter-dimer contacts, A11, A12, A22, and ACN, have been proposed based on information gained primarily from X-ray crystallographic studies of IF protein fragments, cross-linking mass spectrometry analyses of IF protein oligomers, and recent cryo-EM analyses (21–24, 27, 30–35). These four contact types are shown in comparison to those in CreS filaments in Fig. 4*B*, and their general lack of correspondence is elaborated on in the Discussion. In particular, no counterparts of the ACN type (head-to-tail) are observed in our in vitro CreS filament (Fig. 4*B*). However, for eukaryotic IF proteins such as vimentin, it has been proposed that four dimers laterally assemble into an octameric protofibril, of which multiple copies come together in a symmetric way to produce the mature IF, which is often 10 nm wide (13, 27, 29, 36, 37). The two-stranded CreS filament described here consists of four laterally associated CreS dimers that arrange in an octameric manner reminiscent of that in the eukaryotic IF protofibril, as evidenced by previous cross-linkng results and a recent ~7-Å-resolution cryo-EM structure of vimentin IFs (27, 36) (Fig. 4*B*).

### In Vivo Cross-linking Confirms Crescentin Filament Architecture.

To address the in vivo relevance of the CreS filament structure determined in vitro, we probed residue–residue contacts observed in the cryo-EM structure in *C. crescentus* cells using cysteine cross-linking with a thiol-specific and cell-permeable chemical cross-linker, bismaleimidoethane (BMOE, spacer length ~8 Å). Guided by the CreS filament structure described above, we introduced codons of pairs of cysteine residues into *creS*, which was expressed from a low-copy-number plasmid under its native promoter in *C. crescentus* cells, in a *creS* deletion background (*Materials and Methods*). This yielded protein levels of CreS similar to endogenous levels in wt strain CB15N (*SI Appendix*, Fig. S5*A*). Cells expressing wt *creS* or a *creS* mutant this way had a cell curvature comparable to the wt strain, with two exceptions that will be discussed below (*SI Appendix*, Fig. S5*B*). We focused on residue pairs with a Cβ-Cβ distance of 6 to 12 Å at multiple inter-dimer interfaces in both the CreS_sat_ and CreS_wt_ filament structures (*SI Appendix*, Table S4).

**Fig. 5.**
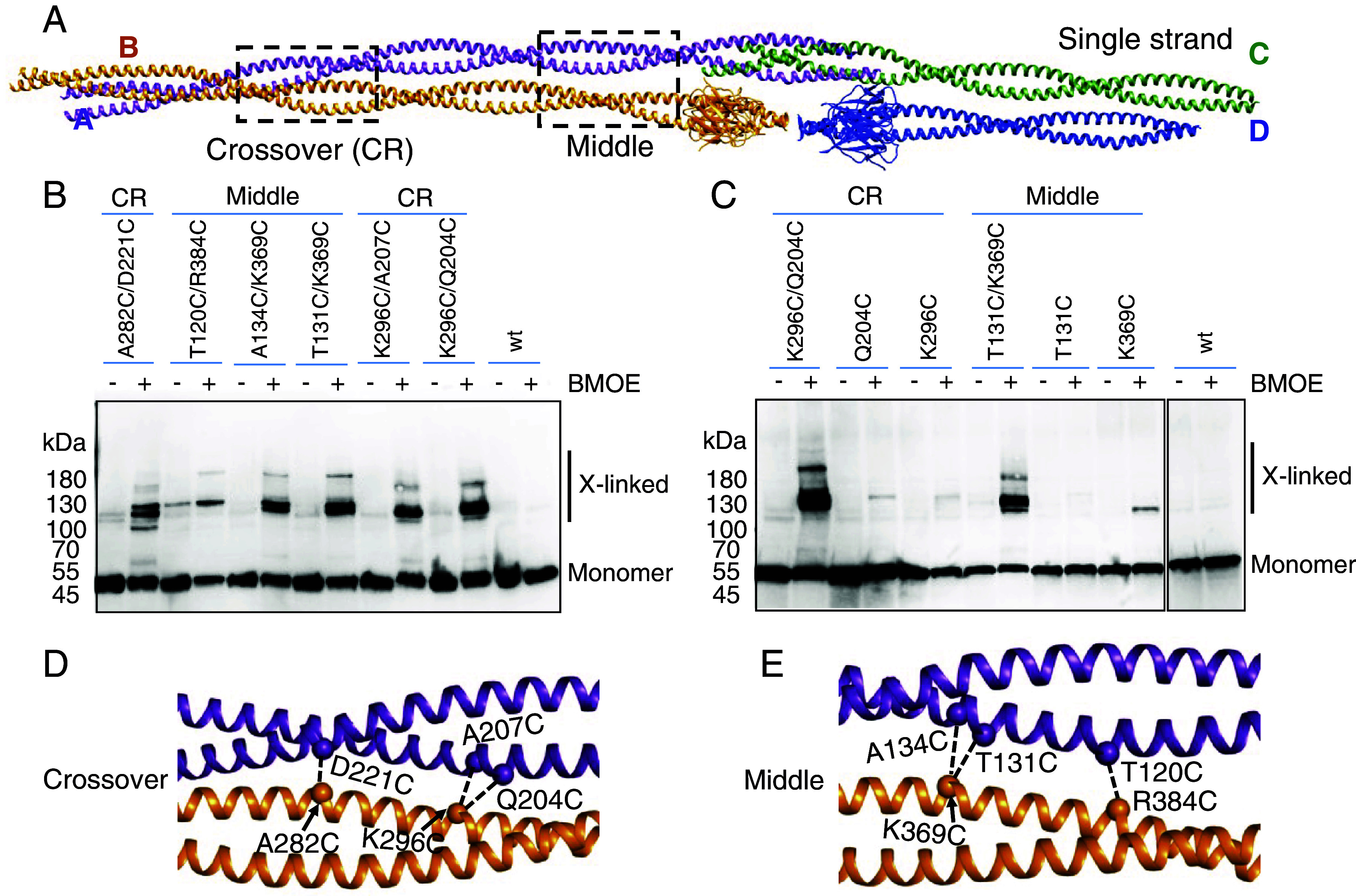
Structure-guided probing of in vivo CreS assembly, with a focus on lateral interactions. (*A*) The single-stranded filament structure of CreS. Dashed rectangles outline the regions in which cysteine substitutions were introduced for in vivo cross-linking. (*B* and *C*) BMOE-dependent cysteine cross-linking of Δ*creS C. crescentus* cells carrying a low-copy-number plasmid expressing *creS* or its cysteine mutants. Reaction products were subjected to Western blotting analysis with a CreS-specific nanobody, NB13. (*D* and *E*) A close-up view of the regions Cross-over (CR, *D*) and Middle (*E*). Cα atoms are shown as spheres. Dashed lines indicate residue–residue pairs probed in *B* and *C*. See also *SI Appendix*, Fig. S6 for similar cross-linking experiments probing longitudinal contacts.

For lateral dimer–dimer interactions (type 3, Fig. 3), we identified BMOE-dependent cross-linking products for at least two residue pairs in each of three regions that are well separated along the elongated inter-dimer interface (Fig. 5 and *SI Appendix*, Fig. S6). In the absence of double-cysteine mutants, we detected no signal or only weak background signal for wt CreS and single cysteine CreS mutants, confirming that cross-linking was largely specific (Fig. 5*C*). The three lateral interaction regions probed include “cross-over” (A282C/D221C, K296C/A207C, and K296C/Q204C), “middle” (T120C/R384C, A134C/K369C, and T131C/K369C), and “NC” (S34C/R419C and A37C/R419C), where NC denotes interactions between the N terminus and C terminus proximal regions (Fig. 5 and *SI Appendix*, Fig. S6).

For longitudinal interactions, we observed BMOE-dependent cross-links between residue pairs near the N termini (E43C/S74C and E57C/E63C, region NN, type 2) and C termini (M443C, region CC, type 1), respectively (*SI Appendix*, Fig. S6). Since all these inter-dimer interaction regions are most likely important for the assembly of individual strands, these results indicate that the single-stranded architecture observed in vitro likely acts as a structural unit for producing CreS assemblies in vivo.

### Subcellular Organization of CreS Filaments.

**Fig. 6.**
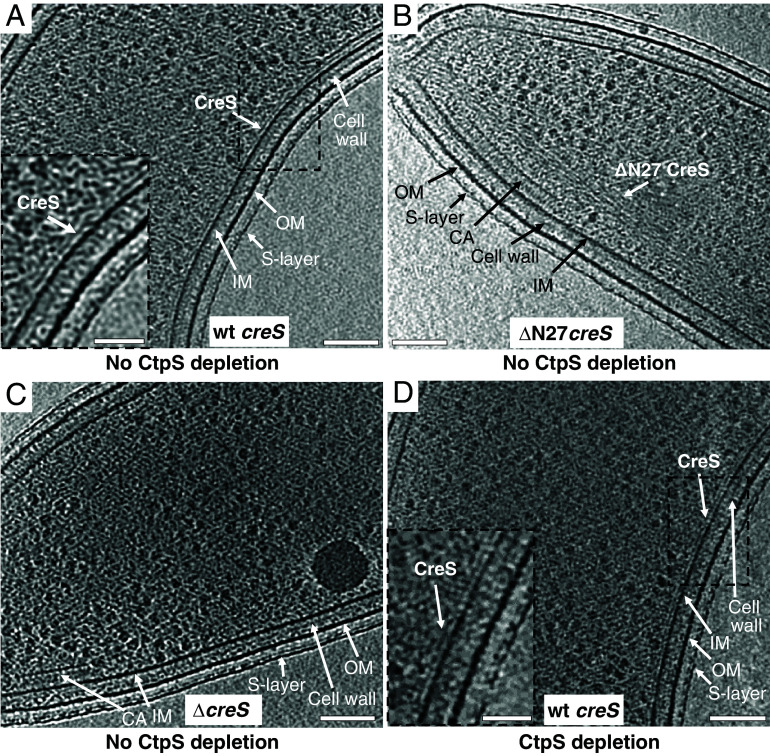
Subcellular organization of CreS filaments in cells. Tomographic slices through wild-type (wt) *C. crescentus* cells with wt *creS* overexpression (*A*, strain CJW1430), Δ*creS* cells with ΔN27 *creS* overexpression (*B*, strain YL006), Δ*creS* cells (*C*, strain LS2813), and Δ*creS* cells with both CtpS depletion and wt *creS* overexpression (*D*, strain YL062). (Scale bar, 100 nm.) The *Insets* (Scale bar, 200 nm.) in *A* and *D* show a close-up view of the area outlined by the dashed rectangle. IM: inner cell membrane; CA: chemosensory array; OM: outer membrane.

The ultrastructure of endogenous CreS assemblies at normal levels in *C. crescentus* has previously been challenging to visualize (5–8, 38, 39). We therefore imaged wt *C. crescentus* cells where wt *creS* was moderately overexpressed using electron cryotomography (cryo-ET). Tomographic reconstructions of these cells revealed a ~4-nm-thick and on average 30- to 40-nm-wide (minimum ~15 nm, maximum ~60 nm wide) structure that lines the inner cell membrane at a distance of ~5 nm and that spans a major portion of the cell’s length on the concave side of the cell (Fig. 6*A*). Consistent with this structure being CreS or CreS-containing filaments, over-production of *creS_ΔN27_*, where the first 27 amino acids were truncated, in a *creS* deletion background led to prominent bundles that detach from the inner cell membrane (Fig. 6*B*). This N-terminal region is positively charged and had previously been shown to be required for attachment of CreS to the inner cell membrane (6, 39). Additionally, similar filamentous densities were not detected in cryo-ET reconstructions of Δ*creS* cells (Fig. 6*C*). Furthermore, CTP synthase (CtpS) is another filament-forming protein that localizes to the inner cell curvature in *C. crescentus* (40). CtpS is known to interact with CreS and acts as a regulator of cell curvature (40). Over-production of wt *creS* in *ΔcreS* cells where *ctpS* expression was suppressed showed a similarly extended structure lining the inner cell curvature (Fig. 6*D*). Thus, CreS can form filamentous structures independent of CtpS along the inner cell curvature in *Caulobacter* cells. This is in agreement with the subcellular localization and low-resolution views of CreS as revealed by diffraction-limited fluorescence microscopy (5–7).

### Functional Requirements.

Having determined the assembly and subcellular organization of CreS, we next addressed the molecular determinants of CreS-dependent cell curvature generation. Ectopic expression of wt *creS* in a heterologous system, *E. coli* strain C41, produced curved cells that are normally straight, as evidenced by phase contrast light microscopy and cryo-EM (*SI Appendix*, Fig. S7 *A*, *E*, *I*, and *L*) (6). Consistent with a role of N-terminal and C-terminal segments in CreS filament assembly, purified CreS_rod_ that contains only the rod domain failed to assemble into long filaments in vitro, and overexpression of *creS_rod_* did not curve *E. coli* cells (*SI Appendix*, Fig. S7 *B*, *F*, *J*, and *L*). Further supporting this idea is that previous mutational analyses showed that deleting each of the four important regions for CreS assembly observed in our structure resulted in altered assembly properties as well as partial or nearly complete loss of cell curvature in *C. crescentus* (9). These include the rod domain (inter-dimer interaction types 3, 4, 5), the N–N region (type 2), residues 121–143 (intended to correspond to the L1 linker region in eukaryotic IF proteins, types 3 and 5), and the C-terminal segment (type 1). In addition, two double-cysteine mutations, E43C/S74C and E57C/E63C, in the aforementioned N–N region (longitudinal inter-dimer interaction type 2) caused cell straightening in *C. crescentus* (*SI Appendix*, Fig. S5*B*). This cell straightening effect caused by double-cysteine mutations can be speculated to be caused by the disruption of interactions necessary for stabilizing the native CreS assembly, introduction of an artificially stabilized non-native CreS assembly, or a combination of both possibilities.

Nevertheless, correct CreS assembly is not the only functional requirement. Similar to previous findings using *C. crescentus* (6), deleting the first 27 amino acids, which are disordered in our cryo-EM maps, or mutating all negatively charged arginine residues to alanine residues in this region (N27RtoA) yielded straight *E. coli* cells without disrupting CreS filament assembly in vitro (*SI Appendix*, Fig. S7 *C*, *D*, *G*, *H*, *K*, and *L*). Thus, the faithful assembly and subcellular organization of CreS on the inner membrane are both indispensable for its function in bacterial cell shape determination.

## Discussion

Since the original discovery of crescentin (5), it has been challenging to clearly define the evolutionary relationship between crescentin and eukaryotic IF proteins. On the one hand, a full-length atomic structure is not currently available for any eukaryotic IF protein and its filamentous assemblies (12, 27), which precludes quantitative structural comparisons. On the other hand, amino acid sequence homology is not a reliable metric when it comes to IF proteins and related proteins. Likewise, tubulin and FtsZ, a bacterial homologue of tubulin, are structural homologues despite very low sequence similarity. More importantly, eukaryotic IF proteins on their own are already diverging in terms of amino acid sequence identity (e.g., 21% between human nestin and human keratin 12 from two different IF types, when only the most well-conserved rod domain is considered) (12). The regularity and relatively simple amino acid compositions of heptad repeats in coiled coil proteins make sequence comparisons potentially not very useful.

To help clarify the evolutionary relationship between crescentin and eukaryotic IF proteins, we have determined the nearly complete atomic structure of crescentin and its filament (Figs. 1–3). To our knowledge, this has so far not been achieved for any other IF or related proteins (12, 28). Coiled coil-containing filaments are often refractory to structure determination by X-ray crystallography and also cryo-EM because of their elongated nature, the smoothness of the filaments, and heterogeneity of the filaments formed. AlphaFold 2 predictions on coiled coil proteins are not as powerful as for globular proteins (19), which is also the case for crescentin (*SI Appendix*, Fig. S1*C*). To circumvent these problems, we obtained a crescentin-binding nanobody that we converted into a megabody for cryo-EM structural studies. Because the megabody bound close to the C terminus of crescentin and all N- and C-terminal ends in the crescentin filament are located in close proximity, this enabled us to obtain a reliable structure of that part at close to 3.3 Å resolution. Further along the filament, atomic modeling was less certain, but the maps indicated that the rod domain of crescentin most likely forms a continuous coiled coil, without flexible linkers. Because we worried about the effect of the conditions used to obtain the filaments, including the use of the megabody, we used site-directed in vivo cysteine cross-linking to verify the obtained structure (Fig. 5 and *SI Appendix*, Fig. S6). Given that we could not find any major discrepancies, we are confident that the obtained structure is a good representation of the structures crescentin forms in cells. Further supporting this notion is that mutations in a number of structurally important regions for CreS assembly identified here lead to an impaired or abolished cellular function of CreS, based on our and previous results (*SI Appendix*, Fig. S7) (9).

The CreS filament structure presented here expands on a collection of previously known similarities between crescentin and IF proteins that led to the categorization of crescentin as an “IF-like” protein (5, 10). While not quantitatively accurate, IF-like is a useful term to distinguish a group of bacterial proteins, including crescentin, from many other coiled coil rich proteins that share with eukaryotic IF proteins only two common properties—their coiled coil dimers act as building blocks to form filamentous assemblies. Although differences exist between crescentin and eukaryotic IF proteins, as described in the Results section, it is evident based on our and previous work (5–9, 13, 25, 27) that they share a range of similarities that collectively define the term “IF-like”:

i) Domain organization—they share a central α-helical rod domain with a comparable length, flanked by head and tail segments or domains.
ii) Basic building block for assembly—they utilize single parallel coiled coil dimers as a basic building block to form higher-order assemblies.
iii) Filament architecture—octameric protofibril assemblies, 10-nm-thick non-polar fibers, and similar modes of inter-dimer interactions (e.g., A12 contact) characterize crescentin and many eukaryotic IF proteins.
iv) Biochemical properties—they are soluble in buffers at high pH and often with low ionic strength, and polymerize at low pH conditions.
v) Polymerization and dynamics—their polymerization requires no co-factors or nucleotides, and their filaments show slow dynamics and turnover (much more static than cytomotive filaments), and extreme polymerization cooperativity (almost no free subunits).
vi) Mechanical properties—the filamentous structures of crescentin and many eukaryotic IF proteins show similar persistence lengths, and are elastic, solid-like, and recover their elasticity after shear.
vii) Cellular function—they have similar structural roles in cell or organelle shape determination (e.g., crescentin that shapes bacterial cells, nuclear lamins that shape the cell nucleus, and neurofilaments that shape the axon of a neuron).

In the cellular context, cryo-ET of cells overexpressing the IF-like crescentin revealed filamentous structures on the concave sides of cells, close to the inner membrane (Fig. 6). Because of earlier misunderstandings (40), we demonstrated that the observed structures are not CTP synthase (CtpS). From these findings, the question arises how the slowly twisting in vitro filaments correspond to the band-like appearance of crescentin in cells. While it is difficult to answer this without further and higher-resolution insights into the structure of the in vivo crescentin assemblies, it is important to note that it has been reported that when crescentin filaments are released from the membrane through the inhibition of MreB or cell wall synthesis, the filaments form helical structures (6). It seems possible that membrane attachment removes some of the twisting that our in vitro structure revealed. The cryo-ET data show crescentin to be close to the membrane (~5 nm in distance). We propose that this distance could slow down or even stop elongasomes moving past as they circle the cells along their short axes to make cell wall (41–43). Slowing down growth of the cell wall on the side of the cell where crescentin is would lead to the crescent shape of the cell.

We confirmed earlier results that the first 27 residues of crescentin are required for its membrane localization (6) (Fig. 6). Despite trying, we have been unable to demonstrate convincingly direct membrane binding of crescentin filaments to lipid membranes or liposomes in vitro. Given the above-mentioned finding that the inhibition of cell wall synthesis or MreB removes crescentin from the membrane (6), it seems likely that membrane attachment, involving the first 27 residues of crescentin, is mediated by another cellular component. One such component could be MreB (7), although AlphaFold 2 is not able to predict a complex between crescentin and MreB in our hands. Possibly also speaking against this idea is the finding that crescentin over-expression in *E. coli* leads to curved cells (6). If another component is needed to localize crescentin to the membrane, then it must be present (and functional) in *E. coli* as well.

Our structure of crescentin and its filament enables us to compare it to what is known about eukaryotic IF proteins and their filaments. It is important to point out again that there is currently no complete atomic structure of any IF protein and in particular of an IF (12, 27). Given the formidable technical difficulties with these proteins, considerable efforts have been spent using a “divide and conquer” approach combining X-ray crystallography, cross-linking, and cryo-EM, to obtain pseudo-atomic models of IF proteins and filaments. Comparing our structure with these models reveals several important similarities and differences (Fig. 4). Crescentin and most IFs form long parallel coiled coil dimers with disordered domains at each end. Vimentin and other cytoplasmic IFs have been shown to form tetramers, two dimers coming together via their coil1 domains (A11 contact) (21–24). No such contact exists in the structure of the crescentin filament. In fact, apart from IF’s A12 contact there does not seem to be much similarity in the way dimers pack into larger fibers between IFs and crescentin. Furthermore, the most important difference between them is the longitudinal contacts. In crescentin, they are exclusively N–N (head-to-head) and C–C (tail-to-tail), and both types of inter-dimer contact have previously been proposed for another bacterial IF-like protein, FilP (44). In contrast, most, if not all, of the current IF models assume ACN contacts that link dimers head-to-tail. It is, however, important to point out that the experimental evidence for ACN contacts is sparse. Their existence seems to be derived from nuclear lamins that form chains of head-to-tail dimers instead of tetramers (35) and from the fact that it is difficult to build larger IF models from A11 tetramers without ACN head-to-tail arrangements.

So, are the significant differences in higher-order filament architecture contradicting the above assertion that crescentin is IF-like? We would argue it is too early for a definitive, structure-based classification. We would need a number of high-resolution filament structures of IF proteins since among themselves they show already significant sequence and length variations, even in the most conserved parts such as the rod domain, N- and C-terminal “consensus motifs” within the rod domain, and the stutter within coil 2 (*SI Appendix*, Fig. S8). It is therefore at least conceivable that IF proteins also form a number of different filament architectures, based on shared principles such as parallel coiled coils. The work presented here provides an important step toward future structure-based classifications.

All IF proteins contain a stutter that inserts into the coiled coil-forming heptad repeat four extra residues. This is thought to not interrupt the coiled coil per se, but to change the superhelical arrangement such that the two helices run more parallel for a short distance. Crescentin also contains a stutter (residues 406–409, *SI Appendix*, Fig. S1*F*), and we have investigated the consequences of removing it by reverting the repeat pattern back to regular heptad repeats by inserting three residues, Ser, Ala, and Thr (SAT), before position 406 (9). While it has been reported that such a modification abolishes crescentin’s ability to curve cells (9), we have found little evidence that it changes its behavior in vitro. The structures of the WT and SAT versions are very similar, except that the SAT version is slightly longer because the helices contain one almost complete extra helical turn. It will need further work to investigate this in more detail. For example, the stutter might be needed to form higher-order assemblies such as the ones seen in cells (Fig. 6).

Going forward, it will be important to figure out whether crescentin filaments locally modify and reduce cell wall synthesis via the elongasome as proposed and how crescentin filaments localize to the inner membrane since we could not reconstitute membrane binding in vitro. Because crescentin filaments are thin and smooth, cellular tomography is unlikely to deliver soon how this is done. Genetic and in vitro approaches will have to be pushed further to provide answers.

There are a number of other IF-like proteins known in bacteria, such as Scy and FilP in *Streptomyces* (10). It will be important to also investigate what structures these proteins form. Revealing the evolutionary relationships between IF and their bacterial counterparts will also require more certainty about the structures of IFs, an area of eukaryotic structural cell biology that needs urgent attention, perhaps using similar nanobody and cryo-EM approaches as pioneered here. But even with the dissimilarities unearthed, between the crescentin filament structure and the current model of IFs, it is intriguing that non-polar octameric filaments made out of long parallel coiled coil dimers characterize both sets of proteins. A picture emerges in which long, extended parallel coiled coil proteins have evolved to produce a variety of filament architectures to generate mechanical and polymerization properties that are required for a multitude of cellular functions, across the tree of life.

## Materials and Methods

### *Caulobacter crescentus* and *Escherichia coli* strains.

*C. crescentus* cells were grown in peptone yeast extract (PYE) media at 30 °C (45). Antibiotics were used when required in liquid (solid) media at the following concentrations: 1 (1) µg/mL and 1 (2) µg/mL for chloramphenicol and oxytetracycline, respectively. For ectopic expression of wild-type (wt) *creS* and its variants in *C. crescentus*, a parental strain as indicated was transformed with an appropriate plasmid, based on either the low-copy-number pCT133 or medium-copy-number pCT155 vector (*SI Appendix*, Table S1). To generate these plasmids, the sequences of *creS* and their native promoter were amplified from strain CB15N and then assembled into the pCT133 or pCT155 backbone using Gibson assembly (New England Biolabs). For protein expression in *E. coli*, an appropriate expression plasmid (*SI Appendix*, Table S1) was electroporated into C41 (DE3) cells unless stated otherwise. Ampicillin and kanamycin were used at 100 µg/mL and 50 µg/mL, respectively, when required. *E. coli* growth and induction were both at 37 °C unless stated otherwise.

For imaging, *C. crescentus* strains were grown overnight in PYE medium with the appropriate antibiotics, sub-cultured, and grown to mid-log phase. Glucose was used at a concentration of 0.2 % (w/v) to suppress protein expression driven by the leaky, xylose-inducible promoter. *E. coli* strains over-expressing CreS constructs were grown overnight in Luria-Bertani (LB) media with ampicillin to stationary phase, sub-cultured, grown to OD_600 nm_ ~0.2, and induced by 0.05 mM IPTG for ~2.5 h.

### Crescentin Expression and Production.

The amino acid sequences of the proteins used in this study are listed in *SI Appendix*, Table S2. Non-tagged wt crescentin (CreS_wt_, GenBank accession identifier: ACL97278.1) was produced in *E. coli* BL21 (DE3) cells carrying a pET29a-based plasmid for CreS_wt_ expression (strain CJW1659, C. Jacobs-Wagner, Stanford University, USA) (9). To produce a “stutter mutant,” CreS_sat_, that has a three amino acid insertion (Ser, Ala, and Thr; SAT) before residue 406 to remove the stutter, strain CJW2045 (C. Jacobs-Wagner) was used (9). Other non-tagged CreS constructs included CreS_rod_ (amino acids 80–444), CreS_ΔN27_ (amino acids 28–457), and CreS_N274RtoA_ (all Arg mutated to Ala in amino acids 1–27). These were constructed using Gibson assembly (New England Biolabs) and employing pFE127 as a parental plasmid, which is a pHis17-based plasmid for expressing CreS-His, full-length CreS followed by GSHHHHHH. The expression and purification of all non-tagged CreS proteins followed the same procedures below. All purification steps were carried out at 4 °C. *E. coli* cells were grown in 2×TY media with kanamycin or ampicillin to reach an OD_600 nm_ of ~0.6 before 1 mM IPTG was added to induce protein expression for 4 h. Cells were then spun down, and pellets were lysed by sonication in 50 mM sodium phosphate buffer, 150 mM NaCl, pH 7.2, 0.1% (v/v) Triton X-100, supplemented with lysozyme, DNase, RNase, and protease inhibitor cocktail (Roche). After centrifugation at 45,000 ×g for 40 min, the supernatant was incubated with 30 g/L of Avicel PH-10 cellulose microspheres (Sigma-Aldrich) overnight (46). The microspheres were washed with 50 mM sodium phosphate buffer, 1 M NaCl, pH 7.2, followed by elution with 50 mM sodium phosphate buffer pH 7.2, 150 mM NaCl, and 40% (w/v) glucose. The eluate was dialyzed against 50 mM CHES, pH 10, and concentrated before being injected onto a HiLoad 16/600 Superdex 200 PG column (Cytiva) for gel filtration in 5 mM Tris-HCl, pH 8. Peak fractions corresponding to CreS were pooled and concentrated using an Amicron Ultra-15 centrifugal filter (30-kDa molecular weight cutoff [MWCO], Millipore) to a final concentration of ~9 g/L.

To purify CreS-His, cells were lysed by sonication in 50 mM Tris-HCl, 300 mM NaCl, and 2 mM tris(2-carboxyethyl)phosphine (TCEP), pH 8 (buffer A), supplemented with lysozyme, DNase, RNase, and protease inhibitor cocktail (Roche). The lysate was cleared by centrifugation at 75,000 ×g for 1 h and filtered before being loaded onto a 5 mL HisTrap HP column (Cytiva). After washing with increased concentrations (0, 20, 50, and 80 mM) of imidazole in buffer A in a stepwise manner, proteins were eluted with 500 mM imidazole in buffer A. Upon concentration, the eluate was further purified via size exclusion chromatography as above, and protein fractions were treated in the same way as mentioned above.

### Expression and Production of NB13 and MB13.

Camelid nanobodies (NBs) were raised and selected against purified CreS_wt_ (in 5 mM Tris-HCl, pH 8) through the commercial service provided by the VIB Nanobody Core, Vrije Universiteit Brussel, Belgium. Twenty-five of 97 NB clones delivered were chosen based on their sequence diversity and antigen binding capacity for another round of selection against CreS filaments at both pH 7 and pH 6.5, using an enzyme-linked immunosorbent assay (ELISA). To identify suitable NB clones for biochemical and structural studies, purified NB and megabodies [MB, a large nanobody derivative described below (14)] were produced for each of the top eight clones selected based on the ELISA data. The binding properties of each protein to purified CreS were examined using size exclusion chromatography (pH 8) and negative staining electron microscopy (pH 7 and pH 6.5). The final selection was clone No. 13, whose megabody, MB13, together with CreS, forms a stable complex at pH 8 that assembles into single, uniform filaments in vitro upon acidification.

To produce the nanobody version of clone No. 13, namely NB13, the non-suppressor *E. coli* strain WK6 (47) was transformed with a phagemid vector pMECS containing the PelB leader sequence, followed by the NB13 sequence and followed by an HA tag and a 6×His tag. For periplasmic expression of the megabody of NB13, MB13, with a C-terminal 6×His tag, a pET-22b-based plasmid was generated using Gibson assembly (New England Biolabs). In this construct, NB13 was grafted onto a circular permutant of the scaffold protein YgjK (*E. coli* K12 glucosidase, 86 kDa) as previously described (14). The resultant plasmid was electroporated into C41 (DE3) cells. For both NB13 and MB13, cells were grown in Terrific Broth with ampicillin, 2 mM MgCl_2_, and 0.1% (w/v) glucose until OD_600 nm_ ~0.6. Protein expression was induced at 25 °C for ~19 h with 1 mM IPTG. All purification steps were carried out at 4 °C unless stated otherwise. Cell pellets were treated with 0.2 M Tris pH 8, 0.5 mM EDTA, 0.5 M sucrose (TES) on ice for 1 h, and four times diluted TES on ice for another 1.5 h. After clearance by centrifugation at 50,000 ×g for 30 min, the resultant periplasmic extract was loaded onto a 5 mL HisTrap HP column (Cytiva). The column was washed sequentially with a) 20 mM Tris pH 8, 900 mM NaCl; b) 20 mM imidazole in buffer B (20 mM Tris-HCl, 50 mM NaCl, pH 8); and c) 50 mM imidazole in buffer B, and eluted with 500 mM imidazole in buffer B. For NB13, the eluate was sufficiently pure and concentrated using an Amicron Ultra-15 centrifugal filter (3 kDa MWCO, Millipore) to a final concentration of ~14 g/L. For MB13, the eluate was concentrated, and further purified via gel filtration using a Superdex 200 Increase 10/300 GL column (Cytiva), equilibrated in 10 mM Tris-HCl and 20 mM NaCl, pH 8. Fractions corresponding to MB13 were pooled and concentrated using a Vivaspin Turbo 15 centrifugal filter (50 kDa MWCO) to a final concentration of ~36 g/L. Purified proteins were flash frozen in liquid nitrogen and stored at −80 °C.

### Electron Microscopy of Negatively Stained Samples.

Purified CreS proteins were diluted into 25 mM PIPES, pH 6.5, to reach a concentration of ~0.2 g/L, and incubated at room temperature (RT) for 15 min to allow polymerization. The resultant sample was applied onto 400-mesh continuous carbon film copper grids (Electron Microscopy Sciences), which were then stained with 2% (w/v) uranyl acetate. Grids were imaged using a Tecnai 12 electron microscope (Thermo Fisher Scientific, TFS) equipped with an Orius CCD camera and operated at 120 kV.

### Single Particle Cryo-EM Sample Preparation.

Purified CreS_wt_ or CreS_sat_ were incubated with MB13 at a molar ratio of 1:4 in 5 mM Tris-HCl pH 8 at 4 °C overnight. The resultant mixture was applied onto a Superose 6 Increase 3.2/300 gel filtration column (Cytiva) in 5 mM Tris-HCl pH 8. Fractions B9 to B5, corresponding to the CreS-MB13 complex (Fig. 1 and *SI Appendix*, Fig. S1), were pooled and concentrated using a Vivaspin 500 centrifugal filter (100 kDa MWCO). The sample was diluted into 25 mM PIPES pH 6.5 to allow for polymerization at RT for 15 min, and then treated with CHAPS through the addition of a 1% (w/v) CHAPS stock solution to reach a final concentration of 0.05% (w/v) at RT for 5 min. This procedure consistently yielded uniform, single CreS filaments in complex with MB13.

For cryo-EM grid preparation, sample aliquots of 3 µL were applied onto UltrAuFoil R2/2 grids (300 mesh, Quantifoil). Grids were blotted for 1 s at 10 °C with 100% relative humidity and immediately plunge frozen into liquid ethane using a Vitrobot Mark IV system (TFS). Movies of MB13-decorated CreS filaments embedded in vitreous ice were collected at liquid nitrogen temperature using a Titan Krios transmission electron microscope, operated at 300 kV and using the program EPU (TFS). For dataset CreS_sat_, movies were collected at a nominal magnification of 81,000× in super-resolution mode using a K3 direct electron detector (Gatan), resulting in a super-resolution pixel size of 0.53 Å/pixel on the specimen level. The total exposure was 53 e^−^/Å^2^ in 2.4 s with a dose rate of 25 e^−^/pixel/s, a frame rate of 60 ms, and a defocus range of 0.4 to 2.6 µm. For dataset Cres_wt_, movies were collected at a nominal magnification of 75,000× using a Falcon 4 camera (TFS), giving a physical pixel size of 1.08 Å/pixel on the specimen level. The total exposure was 34 e^−^/Å^2^ in 10 s with a dose rate of 4 e^−^/pixel/s, a frame rate of 4 ms (EER—electron event representation), and a defocus range of 0.4 to 4.0 µm. Data collection statistics have been summarized in *SI Appendix*, Table S3.

### Image Processing.

The image processing procedures for datasets CreS_wt_ and CreS_sat_ were essentially the same except for slight differences in pixel size and box size (see *SI Appendix*, Table S3 and *Supporting Text* - Methods for details). Movies were corrected for inter-frame motions using MotionCor2 (48). Aligned frames were summed and down-sampled to produce individual micrographs that were used for estimation of contrast transfer function (CTF) parameters using CTFFIND4 (49). Nearly every single filament on the micrographs (e.g., Fig. 1*B*) showed a regular spacing of ~57 nm between neighboring “nodes,” of which each represents a segment of the CreS filament decorated with MB13 molecules. Particles centered on individual nodes were picked from all micrographs that had been denoised for picking purposes using a neural network model pre-trained in Topaz (50, 51). Remaining image processing steps involved the alternate use of Relion 3.1 (52) and cryoSPARC (53). These steps included particle extraction, 2D classification, ab initio 3D reference generation, 3D classification, 3D refinement, Bayesian polishing (54), symmetry expansion (55), and local refinement (see *SI Appendix*, Fig. S2 for details). The resolutions of reconstructions were estimated based on two methods. One was the Fourier shell correlation (FSC) between two independently calculated half maps (gold standard FSC) using an FSC cut-off of 0.143 (56). The other was a model-map FSC between the final EM map and a map computed based on an atomic model (described below), built into and refined against the EM map, using an FSC cut-off of 0.5 (57). All maps were sharpened using a deep learning-based program, DeepEMhancer (58). Local resolution was assessed using Relion (52).

### Atomic Model Building and Refinement.

Detailed procedures are in *SI Appendix*, Supporting Text - Methods. In brief, the reconstructions of CreS_sat_ showed a structure consisting of four partial CreS coiled coil dimers (i.e., two pairs of longitudinally or laterally associated partial dimers) (Fig. 2). The two partial dimers within each longitudinal pair are related by pseudo two-fold symmetry and are held together by interactions between the C termini or between the N termini. Thus, each lateral pair is formed by two segments, the N and C segments, with their N terminus and C terminus oriented toward the pseudo twofold symmetry axis, respectively. For each segment, we computationally determined the amino acid register by aligning the observed map density with the amino acid sequence of CreS_sat_ using a previously described approach (59). In this process, we focused on a fragment of ~20 amino acids that showed the most prominent side chain densities in the highest-resolution map.

For CreS_sat,_ we predicted the coiled coil dimeric structures for the N segment and C segment based on their estimated length using CCFold (60). A homology model of NB13 was generated using SWISS-MODEL (61). These starting atomic models were fitted into the cryo-EM map in Chimera (62), followed by manual model re-building in Coot (63). The atomic coordinates were subsequently refined against the map in real space using Phenix (64, 65), where secondary structure restraints were applied. Multiple cycles of manual model rebuilding and real space refinement improved the fitting of the model into the density map and model geometry. In local regions where the map resolution was not sufficient for an unambiguous secondary structural assignment between a helix and a loop, we assumed that it was helical according to coiled coil and secondary structure predictions.

The omission of three-amino acids (SAT) in CreS_wt_ with respect to CreS_sat_ produces a stutter that locally disrupts the continuity of the dimeric heptad-repeat coiled coil (66). By assuming that the preceding and later segments along the coiled coil have little structural changes upon introduction of the stutter, we built atomic models for CreS_wt_ reconstructions, as guided by the CreS_sat_ structure mentioned above.

The final atomic models were geometrically validated based on the criteria of MolProbity (67). Model statistics have been summarized in *SI Appendix*, Table S3. All figures were generated using Pymol (https://pymol.org/) or Chimera (62).

### Electron Cryotomography (cryo-ET).

*C. crescentus* cells were pelleted at 8,000 ×g for 2 min and resuspended in PYE media to reach an OD_600 nm_ of ~5. The resuspension was mixed with protein A-conjugated 10-nm gold fiducials (BBI Solutions). Aliquots of 2.5 µL sample were applied onto freshly glow-discharged Quantifoil R 3.5/1 Cu/Rh (200 mesh) grids, followed by blotting for 2 to 3 s and plunge freezing into liquid ethane using a Vitrobot Mark IV system (TFS). Specimens were imaged using a Titan Krios transmission electron microscope (TFS), operated at 300 kV and equipped with a BioQuantum imaging filter (Gatan) and a K3 camera (Gatan). Tilt series were collected from −60° to 60° in 2° increments at a nominal magnification of 19,500× with a pixel size of 3.84 Å /pixel using SerialEM (68). The total exposure was ~180 e^−^/Å^2^ with a defocus of 8 to 10 μm. Tilt series were aligned automatically using Batchruntomo in IMOD (69, 70), and tomograms were reconstructed using the SIRT algorithm in TOMO3D (71).

### Mapping of the NB13 Binding Site on Crescentin.

To map the binding site of NB13 on CreS, CreS-His variants were constructed for ectopic expression in C41 (DE3) cells by introducing either a truncation or a substitution into the parental pFE127 plasmid via site-directed PCR-based mutagenesis (KLD enzyme mix, New England Biolabs). Protein expression was induced for 4 h at 37 °C by adding 1 mM IPTG when cell cultures reached OD_600 nm_ ~0.5 in LB with ampicillin. Cell pellets were washed twice with cold PBS and lysed using CelLytic Express (Sigma-Aldrich) with protease inhibitor cocktail (Roche) at RT for 20 min. Purified CreS controls and equivalent OD_600 nm_ units of cleared cell lysates by centrifugation were resolved on a 4 to 20% SDS-polyacrylamide gel (Bio-Rad). In this step, each biological sample was split into two halves, and the two halves were loaded separately on the same gel. For western blotting analysis, one half was immunoblotted with NB13 (7 µg/mL) followed by washing and probing with α-HA-peroxidase (1:1,000, Roche), whereas the other half was probed with α-His-peroxidase only (1:4,000, TFS).

### Whole-cell Cysteine Cross-linking.

*C. crescentus* strains harboring a low-copy-number plasmid for expressing CreS or its mutants were grown overnight in PYE with oxytetracycline, sub-cultured, and grown to OD_600 nm_ 0.5 to 0.6. Control strains, CB15N and CB15N Δ*creS* were grown in the same way without antibiotics. About 1.2 OD_600 nm_ units of cells were spun down at 8,000 ×g for 3 min and then kept on ice for the following steps. Pellets were washed with cold PBS and resuspended in 80 µL of cold PBS, followed by a 10-min reaction upon addition of 2 µL DMSO or bis(maleimido)ethane (BMOE, final concentration 0.5 mM, ~8-Å arm length, TFS) in DMSO. The reaction was quenched by adding 1 µL of β-mercaptoethanol (BME, stock ~2.3 mM). Cells were then lysed using CelLytic Express (Sigma-Aldrich), supplemented with protease inhibitor cocktail (Roche) at RT for 15 min. The suspension was incubated at 70 °C for 5 min in the presence of LDS loading buffer supplemented with 4% (v/v) BME. Samples equivalent to 0.1 OD_600 nm_ units of cells were resolved on a 4 to 20% SDS-polyacrylamide gel (Bio-Rad), and immunoblotted with purified NB13 (16 µg/mL), followed by washing and then probing with α-HA-peroxidase (1:1,000, Roche).

### Light Microscopy.

Cells grown in conditions described above were imaged upon immobilization on 1% agarose pads. Images were acquired using a Nikon Eclipse Ti2 microscope equipped with a Neo sCMOS camera (Andor) and a Nikon Plan APO DIC objective (100×, numerical aperture 1.40). Cell segmentation and cell curvature analysis were performed using MicrobeJ (72) as a plugin in ImageJ (73). The number of cells analyzed was between 114 and 519 for *C. crescentus* strains, and between 140 and 293 for *E. coli* strains. For each strain, mean cell curvature (µm^−1^) and SD (µm^−1^) were computed and presented.

## Supplementary Material

Appendix 01 (PDF)

## Data Availability

The atomic coordinates derived using reconstructions SAT-SB-C2, SAT-SB-C1, SAT-LB-C2, SAT-LB-C1, WT-SB-C2, WT-SB-C1, WT-LB-C2, and WT-LB-C1 have been deposited with the Protein Data Bank [accession numbers 8AFH (74), 8AFE (75), 8AJB (76), 8AHL (77), 8AFM (78), 8AFL (79), 8AIX (80), 8AIA (81). The cryo-EM maps of SAT-SB-C2, SAT-SB-C1, SAT-LB-C2, SAT-LB-C1, WT-SB-C2, WT-SB-C1, WT-LB-C2, and WT-LB-C1 have been deposited with the Electron Microscopy Data Bank (accession numbers EMD-15398 (82), EMD-15395 (83), EMD-15476 (84), EMD-15446 (85), EMD-15402 (86), EMD-15401 (87), EMD-15473 (88), and EMD-15465 (89)]. *SI Appendix*, Table S3 summarizes details.

## References

[1] K. D. Young, The selective value of bacterial shape. Microbiol. Mol. Biol. Rev. **70**, 660–703 (2006). 16959965 10.1128/MMBR.00001-06 PMC1594593

[2] I. V. Surovtsev, C. Jacobs-Wagner, Subcellular organization: A critical feature of bacterial cell replication. Cell **172**, 1271–1293 (2018). 29522747 10.1016/j.cell.2018.01.014 PMC5870143

[3] J. M. Skerker, M. T. Laub, Cell-cycle progression and the generation of asymmetry in Caulobacter crescentus. Nat. Rev. Microbiol. **2**, 325–337 (2004). 15031731 10.1038/nrmicro864

[4] A. Persat, H. A. Stone, Z. Gitai, The curved shape of Caulobacter crescentus enhances surface colonization in flow. Nat. Commun. **5**, 3824 (2014). 24806788 10.1038/ncomms4824 PMC4104588

[5] N. Ausmees, J. R. Kuhn, C. Jacobs-Wagner, The bacterial cytoskeleton: An intermediate filament-like function in cell shape. Cell **115**, 705–713 (2003). 14675535 10.1016/s0092-8674(03)00935-8

[6] M. T. Cabeen , Bacterial cell curvature through mechanical control of cell growth. EMBO J. **28**, 1208–1219 (2009). 19279668 10.1038/emboj.2009.61 PMC2683044

[7] G. Charbon, M. T. Cabeen, C. Jacobs-Wagner, Bacterial intermediate filaments: In vivo assembly, organization, and dynamics of crescentin. Genes Dev. **23**, 1131–1144 (2009). 19417107 10.1101/gad.1795509 PMC2682956

[8] O. Esue, L. Rupprecht, S. X. Sun, D. Wirtz, Dynamics of the bacterial intermediate filament crescentin in vitro and in vivo. PLoS One **5**, e8855 (2010). 20140233 10.1371/journal.pone.0008855 PMC2816638

[9] M. T. Cabeen, H. Herrmann, C. Jacobs-Wagner, The domain organization of the bacterial intermediate filament-like protein crescentin is important for assembly and function. Cytoskeleton (Hoboken) **68**, 205–219 (2011). 21360832 10.1002/cm.20505 PMC3087291

[10] G. H. Kelemen, Intermediate filaments supporting cell shape and growth in bacteria. Subcell Biochem. **84**, 161–211 (2017). 28500526 10.1007/978-3-319-53047-5_6

[11] H. Herrmann, U. Aebi, Intermediate filaments and their associates: Multi-talented structural elements specifying cytoarchitecture and cytodynamics. Curr. Opin. Cell Biol. **12**, 79–90 (2000). 10679360 10.1016/s0955-0674(99)00060-5

[12] P. J. Vermeire , Molecular interactions driving intermediate filament assembly. Cells **10**, 2457 (2021). 34572105 10.3390/cells10092457 PMC8466517

[13] H. Herrmann, U. Aebi, Intermediate filaments: Structure and assembly. Cold Spring Harb. Perspect. Biol. **8**, a018242 (2016). 27803112 10.1101/cshperspect.a018242 PMC5088526

[14] T. Uchanski , Megabodies expand the nanobody toolkit for protein structure determination by single-particle cryo-EM. Nat. Methods **18**, 60–68 (2021). 33408403 10.1038/s41592-020-01001-6 PMC7611088

[15] D. S. Gilbert, B. J. Newby, Neurofilament disguise, destruction and discipline. Nature **256**, 586–589 (1975). 170526 10.1038/256586a0

[16] P. M. Steinert, W. W. Idler, S. B. Zimmerman, Self-assembly of bovine epidermal keratin filaments in vitro. J. Mol. Biol. **108**, 547–567 (1976). 1018318 10.1016/s0022-2836(76)80136-2

[17] W. Renner , Reconstitution of intermediate-sized filaments from denatured monomeric vimentin. J. Mol. Biol. **149**, 285–306 (1981). 7310882 10.1016/0022-2836(81)90303-x

[18] A. Lupas, M. Van Dyke, J. Stock, Predicting coiled coils from protein sequences. Science **252**, 1162–1164 (1991). 2031185 10.1126/science.252.5009.1162

[19] J. Jumper , Highly accurate protein structure prediction with AlphaFold. Nature **596**, 583–589 (2021). 34265844 10.1038/s41586-021-03819-2 PMC8371605

[20] H. Herrmann, M. Haner, M. Brettel, N. O. Ku, U. Aebi, Characterization of distinct early assembly units of different intermediate filament proteins. J. Mol. Biol. **286**, 1403–1420 (1999). 10064706 10.1006/jmbi.1999.2528

[21] A. Aziz , The structure of vimentin linker 1 and rod 1B domains characterized by site-directed spin-labeling electron paramagnetic resonance (SDSL-EPR) and X-ray crystallography. J. Biol. Chem. **287**, 28349–28361 (2012). 22740688 10.1074/jbc.M111.334011 PMC3436525

[22] A. A. Chernyatina, S. Nicolet, U. Aebi, H. Herrmann, S. V. Strelkov, Atomic structure of the vimentin central alpha-helical domain and its implications for intermediate filament assembly. Proc. Natl. Acad. Sci. U.S.A. **109**, 13620–13625 (2012). 22869704 10.1073/pnas.1206836109 PMC3427084

[23] J. Ahn , Structural basis for lamin assembly at the molecular level. Nat. Commun. **10**, 3757 (2019). 31434876 10.1038/s41467-019-11684-x PMC6704074

[24] S. A. Eldirany, M. Ho, A. J. Hinbest, I. B. Lomakin, C. G. Bunick, Human keratin 1/10-1B tetramer structures reveal a knob-pocket mechanism in intermediate filament assembly. EMBO J. **38**, e100741 (2019). 31036554 10.15252/embj.2018100741 PMC6545558

[25] H. Herrmann, U. Aebi, Intermediate filaments: Molecular structure, assembly mechanism, and integration into functionally distinct intracellular Scaffolds. Annu. Rev. Biochem. **73**, 749–789 (2004). 15189158 10.1146/annurev.biochem.73.011303.073823

[26] D. Guzenko, A. A. Chernyatina, S. V. Strelkov, Crystallographic studies of intermediate filament proteins. Subcell Biochem. **82**, 151–170 (2017). 28101862 10.1007/978-3-319-49674-0_6

[27] M. Eibauer , Vimentin filaments integrate low complexity domains in a highly complex helical structure. BioRxiv [Preprint] (2023), 10.1101/2023.05.22.541714 (Accessed 22 May 2023). PMC11189308 38632361

[28] S. A. Eldirany, I. B. Lomakin, M. Ho, C. G. Bunick, Recent insight into intermediate filament structure. Curr. Opin. Cell Biol. **68**, 132–143 (2021). 33190098 10.1016/j.ceb.2020.10.001 PMC7925366

[29] M. S. Weber , Structural heterogeneity of cellular K5/K14 filaments as revealed by cryo-electron microscopy. Elife **10**, e70307 (2021). 34323216 10.7554/eLife.70307 PMC8360650

[30] P. M. Steinert, L. N. Marekov, R. D. Fraser, D. A. Parry, Keratin intermediate filament structure. Crosslinking studies yield quantitative information on molecular dimensions and mechanism of assembly. J. Mol. Biol. **230**, 436–452 (1993). 7681879 10.1006/jmbi.1993.1161

[31] P. M. Steinert, L. N. Marekov, D. A. Parry, Diversity of intermediate filament structure. Evidence that the alignment of coiled-coil molecules in vimentin is different from that in keratin intermediate filaments. J. Biol. Chem. **268**, 24916–24925 (1993). 7693709

[32] P. M. Steinert, L. N. Marekov, D. A. Parry, Conservation of the structure of keratin intermediate filaments: Molecular mechanism by which different keratin molecules integrate into preexisting keratin intermediate filaments during differentiation. Biochemistry **32**, 10046–10056 (1993). 7691168 10.1021/bi00089a021

[33] D. A. Parry, Hard alpha-keratin IF: A structural model lacking a head-to-tail molecular overlap but having hybrid features characteristic of both epidermal keratin and vimentin IF. Proteins **22**, 267–272 (1995). 7479699 10.1002/prot.340220307

[34] N. Mucke , Molecular and biophysical characterization of assembly-starter units of human vimentin. J. Mol. Biol. **340**, 97–114 (2004). 15184025 10.1016/j.jmb.2004.04.039

[35] E. Heitlinger , Expression of chicken lamin B2 in Escherichia coli: Characterization of its structure, assembly, and molecular interactions. J. Cell Biol. **113**, 485–495 (1991). 2016332 10.1083/jcb.113.3.485 PMC2288961

[36] D. A. D. Parry, L. N. Marekov, P. M. Steinert, Subfilamentous protofibril structures in fibrous proteins. J. Biol. Chem. **276**, 39253–39258 (2001). 11495907 10.1074/jbc.M104604200

[37] K. N. Goldie , Dissecting the 3-D structure of vimentin intermediate filaments by cryo-electron tomography. J. Struct. Biol. **158**, 378–385 (2007). 17289402 10.1016/j.jsb.2006.12.007

[38] M. D. Lew , Three-dimensional superresolution colocalization of intracellular protein superstructures and the cell surface in live Caulobacter crescentus. Proc. Natl. Acad. Sci. U.S.A. **108**, E1102–1110 (2011). 22031697 10.1073/pnas.1114444108 PMC3219151

[39] M. T. Cabeen , Mutations in the Lipopolysaccharide biosynthesis pathway interfere with crescentin-mediated cell curvature in Caulobacter crescentus. J. Bacteriol. **192**, 3368–3378 (2010). 20435724 10.1128/JB.01371-09 PMC2897673

[40] M. Ingerson-Mahar, A. Briegel, J. N. Werner, G. J. Jensen, Z. Gitai, The metabolic enzyme CTP synthase forms cytoskeletal filaments. Nat. Cell Biol. **12**, 739–746 (2010). 20639870 10.1038/ncb2087 PMC3210567

[41] J. Dominguez-Escobar , Processive movement of MreB-associated cell wall biosynthetic complexes in bacteria. Science **333**, 225–228 (2011). 21636744 10.1126/science.1203466

[42] E. C. Garner , Coupled, circumferential motions of the cell wall synthesis machinery and MreB filaments in B. subtilis. Science **333**, 222–225 (2011). 21636745 10.1126/science.1203285 PMC3235694

[43] S. van Teeffelen , The bacterial actin MreB rotates, and rotation depends on cell-wall assembly. Proc. Natl. Acad. Sci. U.S.A. **108**, 15822–15827 (2011). 21903929 10.1073/pnas.1108999108 PMC3179079

[44] A. Javadi, N. Söderholm, A. Olofsson, K. Flärdh, L. Sandblad, Assembly mechanisms of the bacterial cytoskeletal protein FilP. Life Sci. Alliance **2**, e201800290 (2019). 31243049 10.26508/lsa.201800290 PMC6599971

[45] B. Ely, Genetics of Caulobacter crescentus. Methods Enzymol. **204**, 372–384 (1991). 1658564 10.1016/0076-6879(91)04019-k

[46] N. Soderholm, A. Javadi, I. S. Flores, K. Flardh, L. Sandblad, Affinity to cellulose is a shared property among coiled-coil domains of intermediate filaments and prokaryotic intermediate filament-like proteins. Sci. Rep. **8**, 16524 (2018). 30410115 10.1038/s41598-018-34886-7 PMC6224456

[47] R. Zell, H. J. Fritz, DNA mismatch-repair in Escherichia coli counteracting the hydrolytic deamination of 5-methyl-cytosine residues. EMBO J. **6**, 1809–1815 (1987). 3038536 10.1002/j.1460-2075.1987.tb02435.x PMC553559

[48] S. Q. Zheng , MotionCor2: Anisotropic correction of beam-induced motion for improved cryo-electron microscopy. Nat. Methods **14**, 331–332 (2017). 28250466 10.1038/nmeth.4193 PMC5494038

[49] A. Rohou, N. Grigorieff, CTFFIND4: Fast and accurate defocus estimation from electron micrographs. J. Struct. Biol. **192**, 216–221 (2015). 26278980 10.1016/j.jsb.2015.08.008 PMC6760662

[50] T. Bepler, K. Kelley, A. J. Noble, B. Berger, Topaz-Denoise: General deep denoising models for cryoEM and cryoET. Nat. Commun. **11**, 5208 (2020). 33060581 10.1038/s41467-020-18952-1 PMC7567117

[51] T. Bepler , Positive-unlabeled convolutional neural networks for particle picking in cryo-electron micrographs. Nat. Methods **16**, 1153–1160 (2019). 31591578 10.1038/s41592-019-0575-8 PMC6858545

[52] S. H. Scheres, RELION: Implementation of a Bayesian approach to cryo-EM structure determination. J. Struct. Biol. **180**, 519–530 (2012). 23000701 10.1016/j.jsb.2012.09.006 PMC3690530

[53] A. Punjani, J. L. Rubinstein, D. J. Fleet, M. A. Brubaker, cryoSPARC: Algorithms for rapid unsupervised cryo-EM structure determination. Nat. Methods **14**, 290–296 (2017). 28165473 10.1038/nmeth.4169

[54] J. Zivanov, T. Nakane, S. H. W. Scheres, A Bayesian approach to beam-induced motion correction in cryo-EM single-particle analysis. IUCrJ **6**, 5–17 (2019). 10.1107/S205225251801463X PMC6327179 30713699

[55] Y. Li , Mechanistic insights into caspase-9 activation by the structure of the apoptosome holoenzyme. Proc. Natl. Acad. Sci. U.S.A. **114**, 1542–1547 (2017). 28143931 10.1073/pnas.1620626114 PMC5320974

[56] S. H. Scheres, S. Chen, Prevention of overfitting in cryo-EM structure determination. Nat. Methods **9**, 853–854 (2012). 22842542 10.1038/nmeth.2115 PMC4912033

[57] P. B. Rosenthal, R. Henderson, Optimal determination of particle orientation, absolute hand, and contrast loss in single-particle electron cryomicroscopy. J. Mol. Biol. **333**, 721–745 (2003). 14568533 10.1016/j.jmb.2003.07.013

[58] R. Sanchez-Garcia , DeepEMhancer: A deep learning solution for cryo-EM volume post-processing. Commun. Biol. **4**, 874 (2021). 34267316 10.1038/s42003-021-02399-1 PMC8282847

[59] Q. Fang , Near-atomic structure of a giant virus. Nat. Commun. **10**, 388 (2019). 30674888 10.1038/s41467-019-08319-6 PMC6344570

[60] D. Guzenko, S. V. Strelkov, CCFold: Rapid and accurate prediction of coiled-coil structures and application to modelling intermediate filaments. Bioinformatics **34**, 215–222 (2018). 28968723 10.1093/bioinformatics/btx551

[61] A. Waterhouse , SWISS-MODEL: Homology modelling of protein structures and complexes. Nucleic Acids Res. **46**, W296–W303 (2018). 29788355 10.1093/nar/gky427 PMC6030848

[62] E. F. Pettersen , UCSF Chimera–A visualization system for exploratory research and analysis. J. Comput. Chem. **25**, 1605–1612 (2004). 15264254 10.1002/jcc.20084

[63] P. Emsley, B. Lohkamp, W. G. Scott, K. Cowtan, Features and development of Coot. Acta Crystallogr. D Biol. Crystallogr. **66**, 486–501 (2010). 20383002 10.1107/S0907444910007493 PMC2852313

[64] P. D. Adams , PHENIX: A comprehensive Python-based system for macromolecular structure solution. Acta Crystallogr. D Biol. Crystallogr. **66**, 213–221 (2010). 20124702 10.1107/S0907444909052925 PMC2815670

[65] P. V. Afonine , Real-space refinement in PHENIX for cryo-EM and crystallography. Acta Crystallogr. D Struct. Biol. **74**, 531–544 (2018). 29872004 10.1107/S2059798318006551 PMC6096492

[66] J. H. Brown, C. Cohen, D. A. Parry, Heptad breaks in alpha-helical coiled coils: Stutters and stammers. Proteins **26**, 134–145 (1996). 8916221 10.1002/(SICI)1097-0134(199610)26:2<134::AID-PROT3>3.0.CO;2-G

[67] V. B. Chen , MolProbity: All-atom structure validation for macromolecular crystallography. Acta Crystallogr. D Biol. Crystallogr. **66**, 12–21 (2010). 20057044 10.1107/S0907444909042073 PMC2803126

[68] D. N. Mastronarde, Automated electron microscope tomography using robust prediction of specimen movements. J. Struct. Biol. **152**, 36–51 (2005). 16182563 10.1016/j.jsb.2005.07.007

[69] D. N. Mastronarde, S. R. Held, Automated tilt series alignment and tomographic reconstruction in IMOD. J. Struct. Biol. **197**, 102–113 (2017). 27444392 10.1016/j.jsb.2016.07.011 PMC5247408

[70] J. R. Kremer, D. N. Mastronarde, J. R. McIntosh, Computer visualization of three-dimensional image data using IMOD. J. Struct. Biol. **116**, 71–76 (1996). 8742726 10.1006/jsbi.1996.0013

[71] J. I. Agulleiro, J. J. Fernandez, Tomo3D 2.0–exploitation of advanced vector extensions (AVX) for 3D reconstruction. J. Struct. Biol. **189**, 147–152 (2015). 25528570 10.1016/j.jsb.2014.11.009

[72] A. Ducret, E. M. Quardokus, Y. V. Brun, MicrobeJ, a tool for high throughput bacterial cell detection and quantitative analysis. Nat. Microbiol. **1**, 16077 (2016). 27572972 10.1038/nmicrobiol.2016.77 PMC5010025

[73] J. Schindelin , Fiji: An open-source platform for biological-image analysis. Nat. Methods **9**, 676–682 (2012). 22743772 10.1038/nmeth.2019 PMC3855844

[74] Y. Liu, J. Löwe, Cryo-EM structure of crescentin filaments (stutter mutant, C2, symmetry and small box). RCSB Protein Data Bank. https://www.rcsb.org/structure/8AFH. Deposited 18 July 2022.

[75] Y. Liu, J. Löwe, Cryo-EM structure of crescentin filaments (stutter mutant, C1 symmetry and small box). RCSB Protein Data Bank. https://www.rcsb.org/structure/8AFE. Deposited 17 July 2022.

[76] Y. Liu, J. Löwe, Cryo-EM structure of crescentin filaments (stutter mutant, C2 symmetry and large box). RCSB Protein Data Bank. https://www.rcsb.org/structure/8AJB. Deposited 28 July 2022.

[77] Y. Liu, J. Löwe, Cryo-EM structure of crescentin filaments (stutter mutant, C1 symmetry and large box). RCSB Protein Data Bank. https://www.rcsb.org/structure/8AHL. Deposited 22 July 2022.

[78] Y. Liu, J. Löwe, Cryo-EM structure of crescentin filaments (wildtype, C2 symmetry and small box). RCSB Protein Data Bank. https://www.rcsb.org/structure/8AFM. Deposited 18 July 2022.

[79] Y. Liu, J. Löwe, Cryo-EM structure of crescentin filaments (wildtype, C1 symmetry and small box). RCSB Protein Data Bank. https://www.rcsb.org/structure/8AFL. Deposited 18 July 2022.

[80] Y. Liu, J. Löwe, Cryo-EM structure of crescentin filaments (wildtype, C2 symmetry and large box). RCSB Protein Data Bank. https://www.rcsb.org/structure/8AIX. Deposited 27 July 2022.

[81] Y. Liu, J. Löwe, Cryo-EM structure of crescentin filaments (wildtype, C1 symmetry and large box). RCSB Protein Data Bank. https://www.rcsb.org/structure/8AIA. Deposited 26 July 2022.

[82] Y. Liu, J. Löwe, Cryo-EM structure of crescentin filaments (stutter mutant, C2 symmetry and small box). Electron Microscopy Data Bank. https://www.ebi.ac.uk/emdb/EMD-15398. Deposited 18 July 2022.

[83] Y. Liu, J. Löwe, Cryo-EM structure of crescentin filaments (stutter mutant, C1 symmetry and small box). Electron Microscopy Data Bank. https://www.ebi.ac.uk/emdb/EMD-15395. Deposited 17 July 2022.

[84] Y. Liu, J. Löwe, Cryo-EM structure of crescentin filaments (stutter mutant, C2 symmetry and large box). Electron Microscopy Data Bank. https://www.ebi.ac.uk/emdb/EMD-15476. Deposited 28 July 2022.

[85] Y. Liu, J. Löwe, Cryo-EM structure of crescentin filaments (stutter mutant, C1 symmetry and large box). Electron Microscopy Data Bank. https://www.ebi.ac.uk/emdb/EMD-15446. Deposited 22 July 2022.

[86] Y. Liu, J. Löwe, Cryo-EM structure of crescentin filaments (wildtype, C2 symmetry and small box). Electron Microscopy Data Bank. https://www.ebi.ac.uk/emdb/EMD-15402. Deposited 18 July 2022.

[87] Y. Liu, J. Löwe, Cryo-EM structure of crescentin filaments (wildtype, C1 symmetry and small box). Electron Microscopy Data Bank. https://www.ebi.ac.uk/emdb/EMD-15401. Deposited 18 July 2022.

[88] Y. Liu, J. Löwe, Cryo-EM structure of crescentin filaments (wildtype, C2 symmetry and large box). Electron Microscopy Data Bank. https://www.ebi.ac.uk/emdb/EMD-15473. Deposited 27 July 2022.

[89] Y. Liu, J. Löwe, Cryo-EM structure of crescentin filaments (wildtype, C1 symmetry and large box). Electron Microscopy Data Bank. https://www.ebi.ac.uk/emdb/EMD-15465. Deposited 26 July 2022.

