# Supplementary material for: Filament structure and subcellular organization of the bacterial intermediate filament–like protein crescentin
Source: Proc Natl Acad Sci U S A. 2024 Feb 7;121(7):e2309984121. doi: 10.1073/pnas.2309984121 (PMC10873595; doi:10.1073/pnas.2309984121)
Supplement: Supplementary file 1 — Appendix 01 (PDF) [file pnas.2309984121.sapp.pdf]

PNAS

Supporting Information for

Filament structure and subcellular organization of the bacterial intermediate filament-like protein crescentin

Yue Liu, Fusinita van den Ent, Jan Löwe

Corresponding author: Jan Löwe, email jyl@mrc-lmb.cam.ac.uk

This PDF file includes:

Supporting Text - Methods
Figures S1 to S8
Tables S1 to S4
SI References

SUPPORTING TEXT - METHODS

Image processing (detailed methods). The image processing procedures for datasets $CreS_{wt}$ and $CreS_{sat}$ were essentially the same except for slight differences in pixel size and box size (see **Table S3** and **Fig. S2** for details). The following description focuses on dataset $CreS_{sat}$. Movies were corrected for inter-frame motions using MotionCor2 [1]. Aligned frames were summed and down-sampled to produce individual micrographs (1.06 Å/pixel) that were used for estimation of contrast transfer function (CTF) parameters using CTFFIND4 [2]. Nearly every single filament on the micrographs (e.g., **Fig. 1B**) showed a regular spacing of ~57 nm between neighbouring “nodes”, of which each represents a segment of the CreS filament decorated with MB13 molecules. About 870,000 particles centred on individual nodes were picked from all micrographs that had been denoised for picking purposes using a neural network model pre-trained in Topaz [3, 4].

The following steps involved the alternate use of Relion 3.1 [5] and cryoSPARC [6]. Particles were first extracted with a box size of 100 pixels and 4.24 Å pixel size (Fourier cropping throughout the processing). About 690,000 particles were retained after reference-free 2D classification where low-quality particles were identified and removed. Six *ab initio* 3D references were then generated, and the best resolved reconstruction was used as a starting reference for global particle alignments. Particles were subsequently re-extracted with a box size of 200 pixels and 2.12 Å pixel size. Heterogenous refinement in 3D with four classes resulted in one class of particles that showed partial occupancy of MB13 molecules and those were discarded. Visual inspection of the map reconstructed using the remaining ~600,000 particles suggested the presence of a two-fold symmetry axis perpendicular to the filament axis. Thus, the poses of these particles were then refined where C2 symmetry was imposed. Next, to produce high resolution reconstructions, particles were re-centred and re-extracted with 1.325 Å pixel size and with a box size of 320 pixels, hereafter referred to as “small box” (SB). In parallel, given that a box size of 400 Å is not sufficient to reveal the structure of a complete CreS molecule, another set of particle images were extracted with 1.59 Å pixel size and a box size of 600 pixels, hereafter referred to as “large box” (LB). The following procedures were applied independently to both sets of particle images. After 3D refinement and Bayesian polishing [7], the densities of scaffold proteins (i.e., YgjK) were subtracted from original particle images. The resultant particle images, which essentially represent nanobody-bound CreS filament segments were subjected to local refinement with C2 symmetry, yielding the reconstruction SAT-SB-C2 for set SB (SAT-LB-C2 for set LB, see **Table S3**). To improve map resolutions, particles were expanded by the C2 symmetry [8], and the densities corresponding to one of two symmetry-related copies were subtracted from images. Subsequent local refinement with C1 symmetry led to a higher resolution reconstruction, SAT-SB-C1 for set SB (SAT-LB-C1 for set LB). The resolutions of reconstructions were estimated based on two methods. One was the Fourier Shell Correlation (FSC) between two independently calculated half maps (Gold Standard FSC) using an FSC cut-off of 0.143 [9]. The other was a model-map FSC between the final EM map and a map computed based on an atomic model (described below),

built into and refined against the EM map, using an FSC cut-off of 0.5 [10]. All maps were sharpened using a deep learning-based programme, DeepEMhancer [11]. Local resolution was assessed using Relion [5].

Atomic model building and refinement (detailed methods). The reconstructions SAT-SB-C1 and SAT-LB-C1 showed a structure consisting of four partial CreS coiled coil dimers (i.e., two pairs of longitudinally or laterally associated partial dimers) (**Fig. 2**). The two partial dimers within each longitudinal pair are related by pseudo two-fold symmetry and are held together by interactions between the C-termini or between the N-termini. Thus, each lateral pair is formed by two segments, the N- and C-segments, with their N-terminus and C-terminus oriented towards the pseudo two-fold symmetry axis, respectively. For each segment, we computationally determined the amino acid register by aligning the observed map density with the amino acid sequence of $CreS_{sat}$ using a previously described approach [12]. In this process, we focused on a fragment of ~20 amino acids that showed the most prominent side chain densities in SAT-SB-C1. Briefly, every amino acid position in the fragment was assigned a number among 0-6 based on the estimated size of the side chain density by visual inspection. The resultant sequence of numbers was searched against that of the entire amino acid sequence of $CreS_{sat,}$ according to a scoring matrix, where a mismatch of a small residue in the sequence with the observed large side chain density at a given position was penalized. The amino acid register determined *in silico* this way was further supported by several lines of evidence. First, the resultant amino acid sequence fitted well into the observed side chain density (e.g., **Fig. S3**), as judged by visual inspection. Second, the binding region of NB13 on CreS was mapped to residues 413-433 (**Fig. 2A**), consistent with the assigned amino acid register for the C segment. Third, whole cell cysteine crosslinking revealed residue-residue contacts that were consistent with the atomic model of the single-stranded structure (**Figs. 5 & S6**). In particular, laterally associated inter-dimer residue-residue contacts supported the assigned amino acid register for the N segment.

To interpret the highest resolution reconstruction SAT-SB-C1, we predicted the coiled coil dimeric structures for the N segment and C segment based on their estimated length using CCFold [13]. A homology model of NB13 was generated using SWISS-MODEL [14] with the structure of a nanobody (NB-ALFA) against the ALFA-tag (PDB entry 6I2G) as a template. These starting atomic models were fitted into the cryo-EM map in Chimera [15], followed by manual model re-building in Coot [16]. In this process, the orientation of NB13 was initially determined based on the β-strands identified in the map and was similar to that of NB-ALFA when complexed with the ALFA-tag that forms an α-helix. The atomic coordinates were subsequently refined against the map in real space using Phenix [17, 18], where secondary structure restraints were applied. Multiple cycles of manual model rebuilding and real space refinement improved the fitting of the model into the density map and model geometry. To interpret the reconstruction SAT-LB-C1, we expanded the atomic model for SAT-SB-C1 by fitting predicted structures of the remaining regions of $CreS_{sat}$. In local regions where the map resolution was not sufficient for an unambiguous secondary structural assignment

between a helix and a loop, we assumed that it was helical according to coiled coil and secondary structure predictions. The expanded atomic model was rebuilt and refined using the same procedures described above. For each of the C2 reconstructions, SAT-SB-C2 and SAT-LB-C2, the atomic model for their C1 counterpart and the C2 symmetry related copy were fitted separately into the map as rigid bodies, and then combined into a single atomic model. Per-atom real space refinement of the resultant atomic model was not performed due to the low-resolution nature of these reconstructions compared with their respective C1 counterparts.

The omission of three-amino acids (SAT) in $CreS_{wt}$ with respect to $CreS_{sat}$ produces a stutter that locally disrupts the continuity of the dimeric heptad-repeat coiled coil [19]. Thus, it is reasonable to assume that the preceding and later segments along the coiled coil have little structural changes upon introduction of the stutter. In line with this, we found that the overall arrangement of $CreS_{wt}$ dimers in the filament is similar to that observed for $CreS_{sat}$ (**Fig. 2**). We determined the amino acid register for the C segment using NB13 molecules as a spatial reference and for the N segment by assuming that $CreS_{wt}$ and $CreS_{sat}$ share the same lateral, inter-dimer interactions between the N-proximal region and C-proximal region. To interpret the reconstruction WT-SB-C1, we used the $CreS_{sat}$ structure as a starting atomic model and replaced the segment corresponding to the stutter region in $CreS_{wt}$ with a sequence adjusted, stutter-containing fragment in vimentin (PDB entry 1GK4). The resultant atomic model was subjected to multiple rounds of model rebuilding and real space refinement against the map, as mentioned above. For the reconstruction WT-LB-C1, the atomic model for WT-SB-C1 was expanded with appropriate segments from the $CreS_{sat}$ structure, followed by model rebuilding and refinement. The atomic model for each of the two C2 reconstructions, WT-SB-C2 and WT-LB-C2, was generated using the aforementioned rigid body fitting approach.

The final atomic models were geometrically validated based on the criteria of MolProbity [20]. Model statistics have been summarized in **Table S3**. All figures were generated using Pymol (https://pymol.org/) or Chimera [15].

SUPPORTING FIGURES

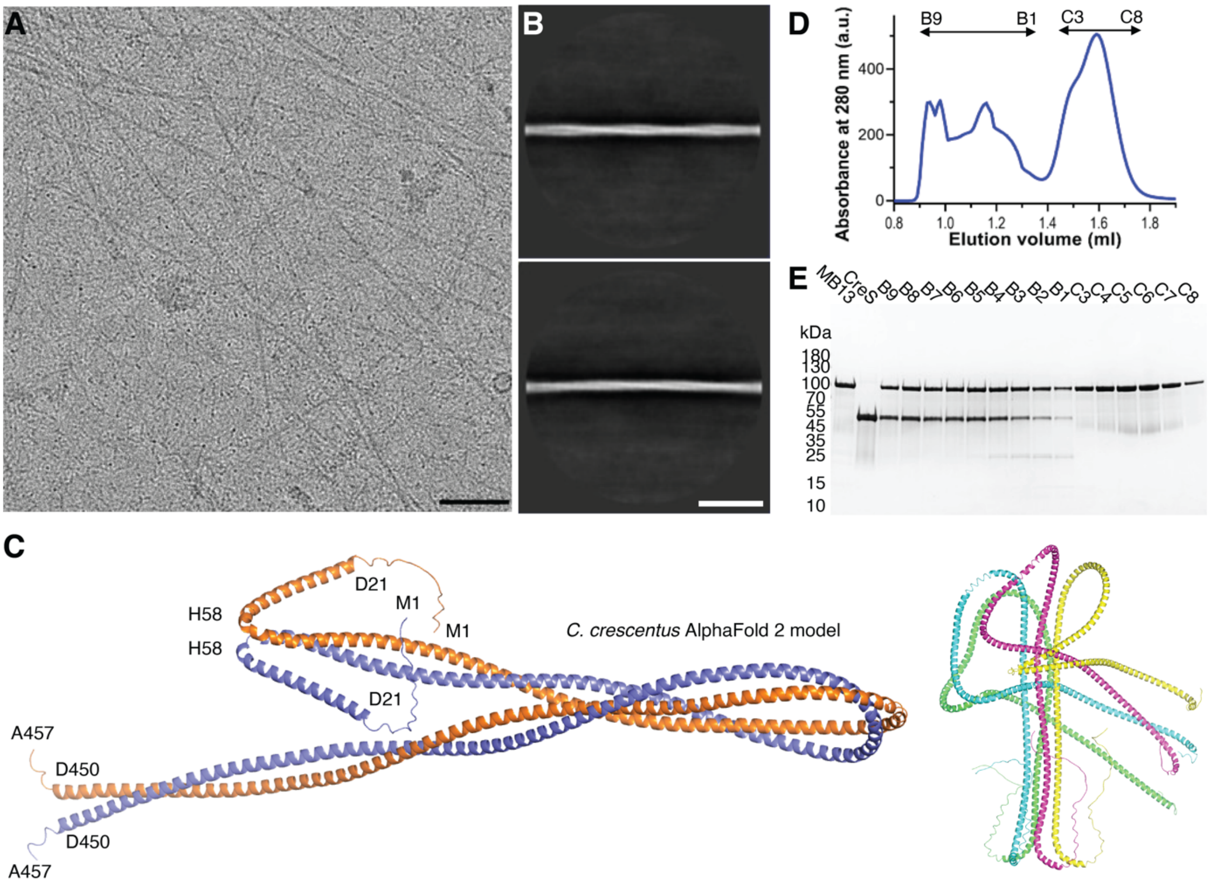

F

```
         1234567890123456789012345678901234567890123456789012345678901234567890

  1      MRLLSKNSRETKNGKPTVLGDEARAEAMQHQIESTQAIGQRYETIHGGLDSIGRVMEHLKAIEPLIAEIR
  1      ......................................................................

 71      GPVSQEFEARRAEHAELIAVRANLDQAQRQIALIQAEEREVSARLAAAETALGESDARRQTQDAALEDNA
 71      ..............fgabcdefgabcdefgabcdefgabcdefgabcdefgabcdef.............

141      LEIDRLRNALLQSDLKVSSLDASLRDATARIEHLVQDVEGLRVQAQDIDARRGDAEAALARANQDNALLG
141      .gabcdefgabcdefgabcdefgabcdefgabcdefgabcdefgabcdefgabcdefgabcdefgabcde

211      EEAATLKKRVDQAGLDLARLSRIETDLEAQLAAERARVQAVENALAAHQADSGRTIRGLESQVEANRAEI
211      fgabcdefgabcdefgabcdefgabcdefgabcdefgabcdefgabcdabcdefgabcdefgabcdefga

281      SALQTRLETATGRADKLEEMNGQISARLADSSAQQKAVERRAGDLNVALERALDRIRALEEEADGLRQRH
281      bcdefgabcdefgabcdefgabcdefgabcdefgabcdefgabcdefgabcdefgabcdefgabcdefga

351      AGVDTARATAIERADQLAKSAVAQEKALKRAEERAQQLRARLDAMQEAQDQVRRDHEAKIAELQATIERL
351      bcdefgabcdefgabcdefgabcdefgabcdefgabcdefgabcdefgabcdefgabcgabcdefgabcd

421      TSEAALAEGALEAARRDRSRLQMALLGASDGDVAASA
421      efgabcdefgabcdefgabcdefgab...........
```

Figure S1. CreS$_{wt}$ assembles into two-stranded filaments *in vitro*. (**A**) Cryo-EM micrograph of single filaments of CreS$_{wt}$ formed at pH 7. (**B**) Two-dimensional (2D) class averages show the presence of two intertwined strands. Scale bar: 100 nm (**A**) and 50 nm (**B**). Megabody MB13 forms a complex with CreS$_{wt}$. (**C**) Size exclusion chromatography (SEC) profile of CreS$_{wt}$, preincubated with an excess of MB13 (1:4 molar ratio) at pH 8. (**D**) SDS-PAGE analysis of SEC fractions. Fractions B9 to B5 were pooled and used for EM analysis. MB13 top, CreS$_{wt}$ bottom bands. **(E)**

Left: annotated AlphaFold 2 model of CreS dimer. Right: AlphaFold 2 model of CreS tetramer. **(F)** PairCoil2 coiled coil prediction of crescentin. Deviations from the canonical heptad repeat are highlighted in yellow.

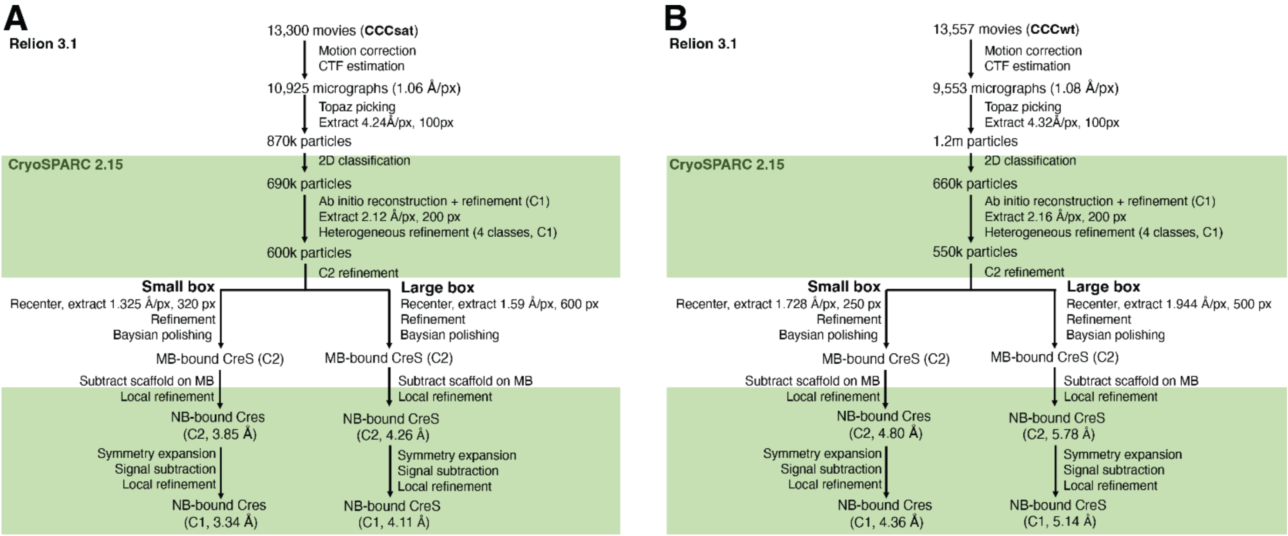

Figure S2. Cryo-EM structure determination of CreS. Image processing workflow used for $CreS_{sat}$ (**A**) and $CreS_{wt}$ (**B**).

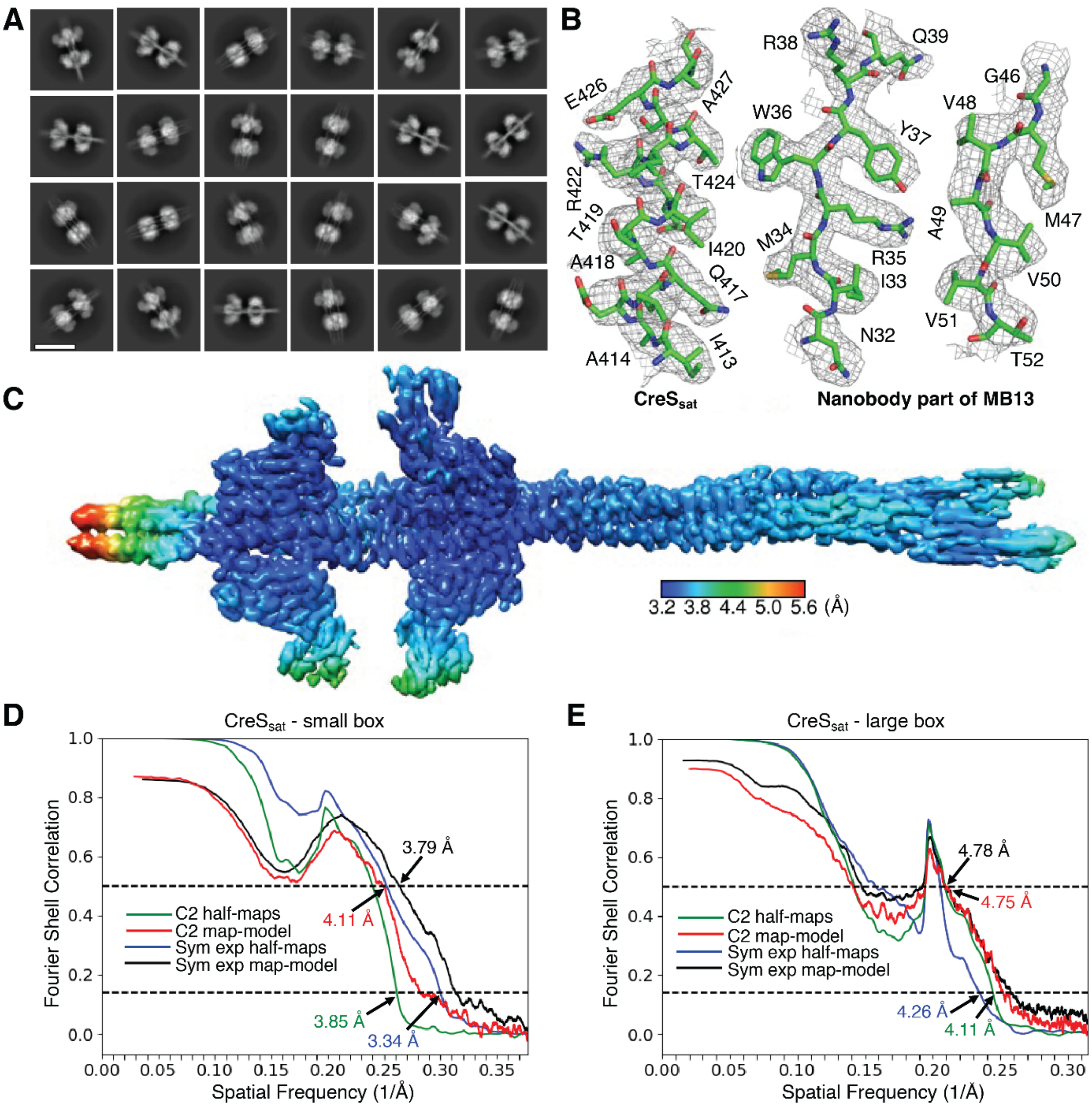

Figure S3. Cryo-EM analysis of CreS$_{sat}$ filaments in complex with megabody MB13. Related to **Figure 2**. (**A**) 2D class averages of individual nodes formed by MB13 and CreS$_{sat}$ in different orientations. (**B**) Typical cryo-EM map densities for CreS$_{sat}$ and MB13, with the fitted atomic model superimposed. (**C**) Local resolution of the symmetry-expanded map reconstructed with a box size of 424 Å. FSC-based resolution estimation for maps reconstructed using a box size of 424 Å (**D**, small box) and 960 Å (**E**, large box).

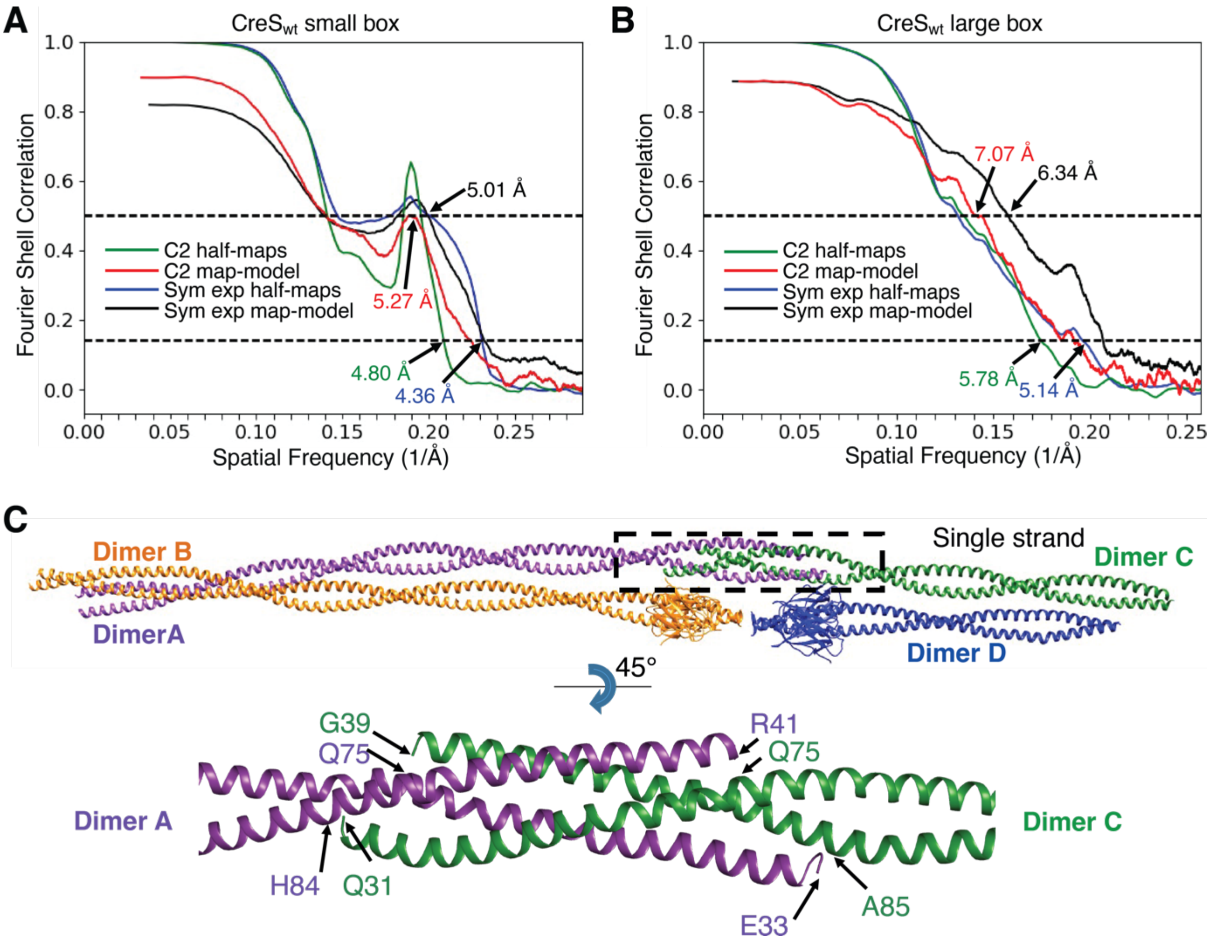

Figure S4. (A, B) Cryo-EM analysis of CreS$_{wt}$ filaments in complex with megabody MB13. Related to **Fig. 2.** Resolution estimation for maps reconstructed using a box size of 432 Å (**A**, small box) and 972 Å (**B**, large box) based on FSC curves. **(C)** The single-stranded filament structure of CreS (upper part). A dashed rectangles defines the limit of the close-up view in the N-N contact region between longitudinal dimers, where residues at the ends of the segments involved are labelled (lower part).

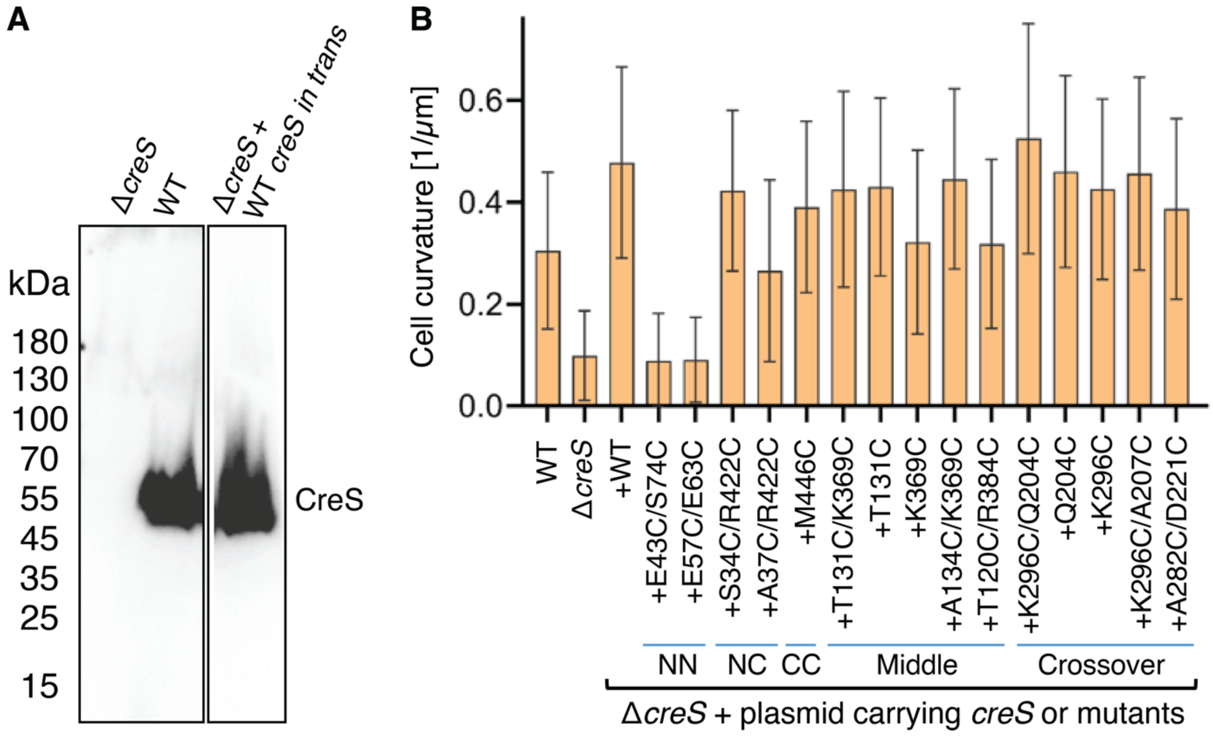

Figure S5. Characterization of *C. crescentus* cells carrying a *creS*-expressing plasmid. (**A**) Western blotting analysis of CreS protein levels in *C. crescentus* strains. Production of CreS from a low-copy-number plasmid under its native promoter in a *creS* deletion background yields a similar protein level as that in the wt strain. (**B**) Quantification of cell curvature of *C. crescentus* strains used for cysteine crosslinking experiments (as in **Figs 5 & S6**). The mean curvature values are shown, and error bars represent standard deviations. The number of cells analysed for each strain was between 114 and 591.

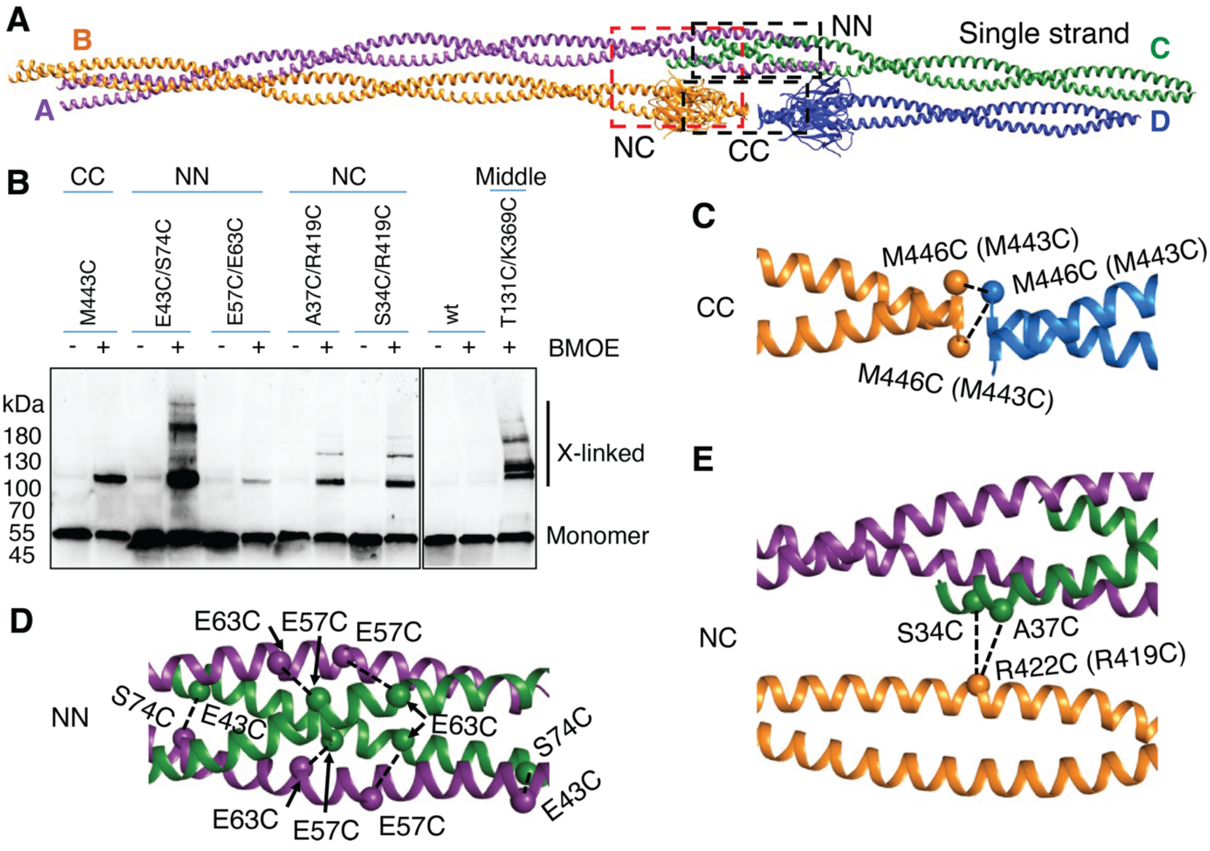

Figure S6. Structure-guided probing of *in vivo* CreS assembly, with a focus on longitudinal interactions. (A) The single-stranded structure of CreS. Dashed rectangles outline the regions where cysteine substitutions were introduced for *in vivo* crosslinking. (**B**) BMOE-mediated cysteine crosslinking of Δ*creS C. crescentus* cells carrying a low-copy-number plasmid expressing *creS* or its cysteine mutants. Reaction products were analyzed as described in **Fig. 5B**. (**C-E**) A close-up view of the regions CC (**C**), NN (**D**), and NC (**E**). Cα atoms are shown as spheres. Dashed lines indicate residue-residue pairs probed in **B**. As in **Fig. 1**, residues in **C** and **E** are numbered based on $CreS_{sat}$, whereas residue numbers in parentheses are according to $CreS_{wt}$.

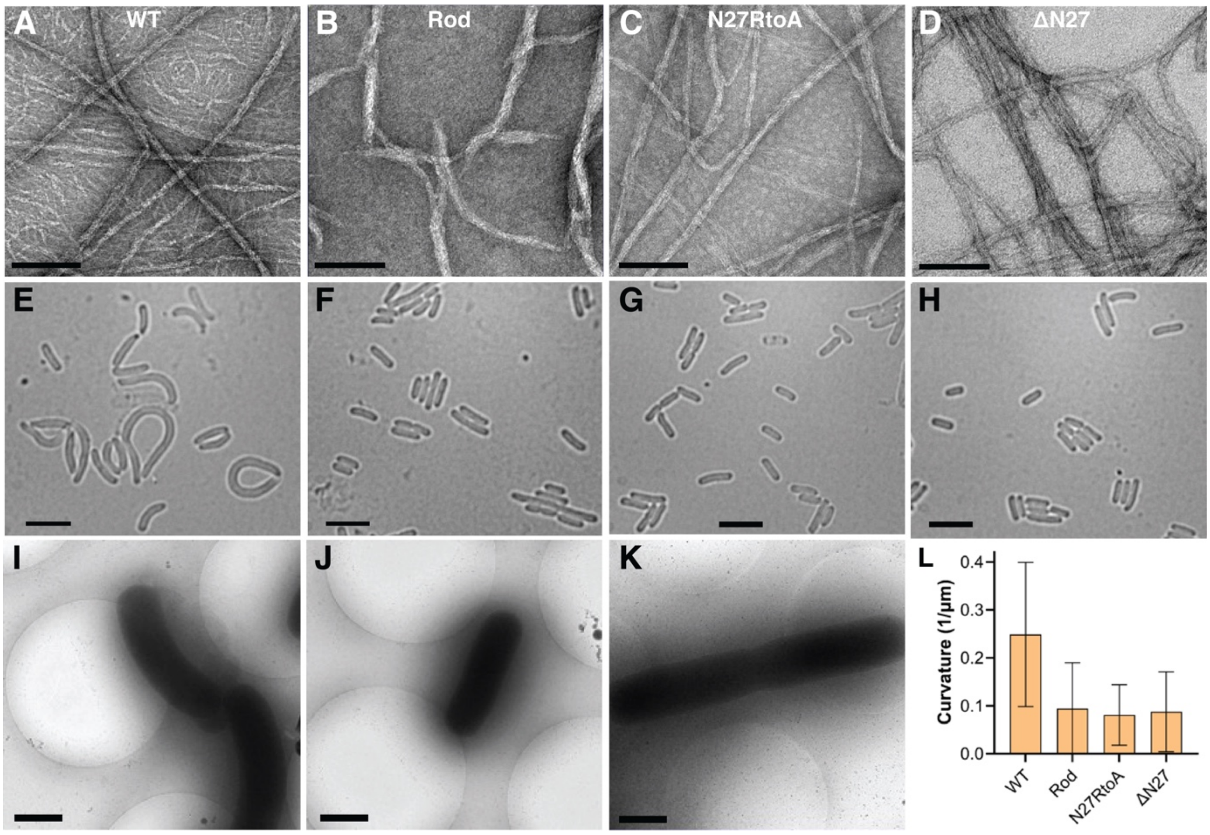

Figure S7. The N-terminal residues 1-27 of CreS are required for its cell shape function in *E. coli*. (**A-D**) EM micrographs of negatively stained filaments formed *in vitro* using purified $CreS_{wt}$ (**A**), $CreS_{rod}$ (**B**), $CreS_{N27RtoA}$, where all Arg residues were substituted with an Ala in the N-terminal 27 amino acids (**C**), and $CreS_{\Delta N27}$ (**D**). Scale bar: 100 nm. (**E-J**) Typical phase contrast images of *E. coli* cells overexpressing $CreS_{wt}$ (**E**), $CreS_{rod}$ (**F**), $CreS_{N27RtoA}$ (**G**), and $CreS_{\Delta N27}$ (**H**). Scale bar: 5 μm. (**I-K**) Typical cryo-EM images of *E. coli* cells overexpressing $CreS_{wt}$ (**I**), $CreS_{rod}$ (**J**), and $CreS_{N27RtoA}$ (**K**). Scale bar: 1 μm. (**L**) Quantification of cell curvature based on phase contrast images (corresponding to panels **E-J**). Shown are the mean curvature values with error bars representing standard deviations. The numbers of cells analysed for each strain were between 140 and 293. Protein expression was induced by 0.05 mM IPTG for ~2.5 h.

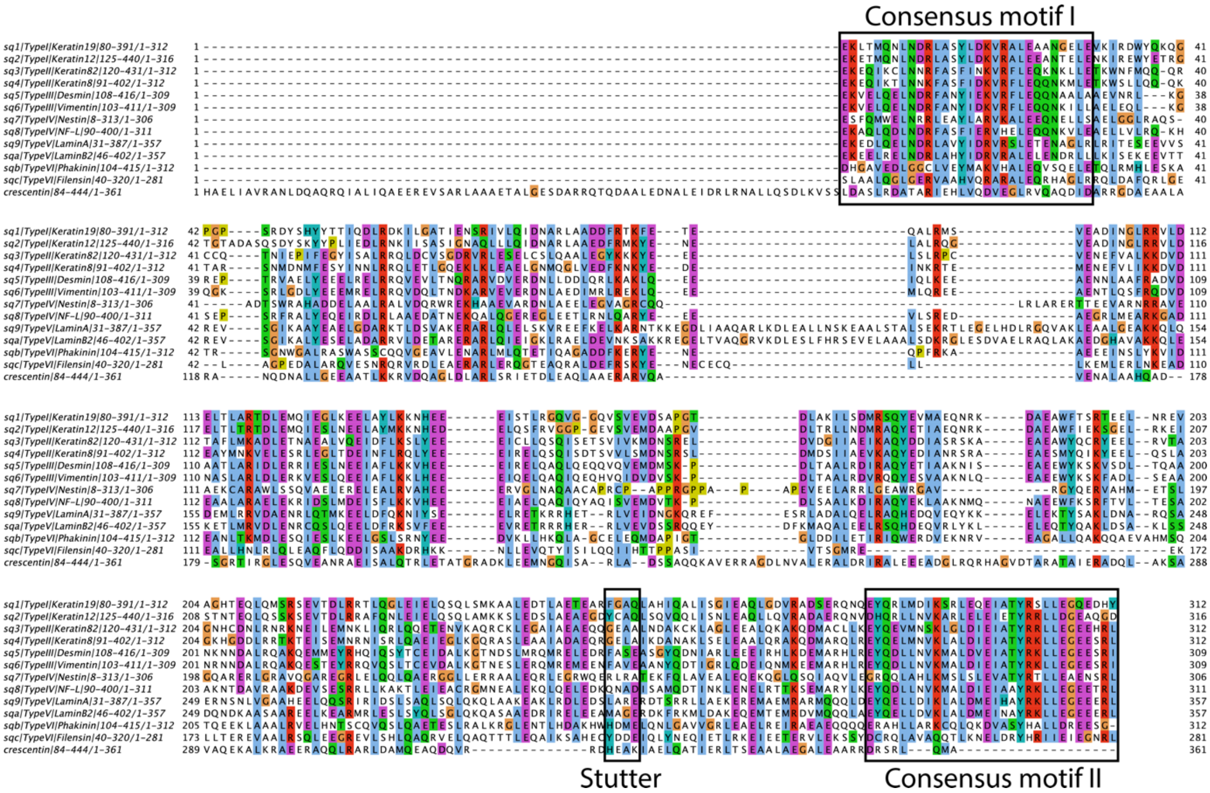

Figure S8. ClustalW multiple sequence alignment of IF proteins and crescentin. Features conserved amongst IF proteins have been highlighted and diverge more for phakinin and filensin. The coiled coil heptad repeat dominates the alignment in the rod domain. The crescentin alignment is questionable, for example around Consensus motif I because of overall low similarity and crescentin being longer, but the position of the stutter is conserved across all sequences.

SUPPORTING TABLES

Table S1. Strains and plasmids

Name	Description/Genotype	Source
**Strains**		
E. coli		
BL21 (DE3)	F– *ompT hsdSB (rB- mB-) gal dcm* (DE3)	NEB
C41 (DE3)	F– *ompT hsdSB (rB- mB-) gal dcm* (DE3)	Sigma-Aldrich
WK6	F' *lacIq* Δ(*lacZ*)M15 *proA+B+* Δ(*lac-proAB*) *galE rpsL*	VIB
CJW1659	BL21 (DE3) expressing $CreS_{wt}$ (non-tagged)	[23]
CJW1661	C41 (DE3) expressing $CreS_{wt}$ (non-tagged)	[23]
CJW2045	BL21 (DE3) expressing $CreS_{sat}$ (insertion of three amino acids (Ser, Ala, SAT, at position 406)	[23]
C. crescentus (vibrioides)		
CB15N "wt" strain in this study	Synchronizable strain of CB15	[24]
LS2813	CB15N Δ*creS*	[25]
ZG216	CB15N Δ*creS ctpS*1-400 P_{xylX}-*ctpS* (*ctpS* truncated to first 400 bp and another *ctpS* under a xylose inducible promoter on chromosome)	[26]
CJW1430	CB15N + pJS14-based medium-copy-number plasmid for expressing CreS under its native promoter	[27]
YL010	CB15N Δ*creS* + pCT133-P_{cres}-$CreS_{wt}$	This study
YL006	CB15N Δ*creS* + pCT133-P_{cres}-$CreS_{\Delta N27}$	This study
YL012	CB15N Δ*creS* + pCT133-P_{cres}-$CreS_{M443C}$	This study
YL017	CB15N Δ*creS* + pCT133-P_{cres}-$CreS_{E43C+S74C}$	This study
YL018	CB15N Δ*creS* + pCT133-P_{cres}-$CreS_{E57C+E63C}$	This study
YL019	CB15N Δ*creS* + pCT133-P_{cres}-$CreS_{R419C+S34C}$	This study
YL020	CB15N Δ*creS* + pCT133-P_{cres}-$CreS_{R419C+A37C}$	This study
YL022	CB15N Δ*creS* + pCT133-P_{cres}-$CreS_{K369C+T131C}$	This study
YL023	CB15N Δ*creS* + pCT133-P_{cres}-$CreS_{K369C+A134C}$	This study
YL024	CB15N Δ*creS* + pCT133-P_{cres}-$CreS_{R384C+T120C}$	This study
YL025	CB15N Δ*creS* + pCT133-P_{cres}-$CreS_{K296C+Q204C}$	This study
YL026	CB15N Δ*creS* + pCT133-P_{cres}-$CreS_{K296C+A207C}$	This study
YL027	CB15N Δ*creS* + pCT133-P_{cres}-$CreS_{A282C+D221C}$	This study
YL031	CB15N Δ*creS* + pCT133-P_{cres}-$CreS_{Q204C}$	This study
YL032	CB15N Δ*creS* + pCT133-P_{cres}-$CreS_{K296C}$	This study
YL033	CB15N Δ*creS* + pCT133-P_{cres}-$CreS_{T131C}$	This study
YL034	CB15N Δ*creS* + pCT133-P_{cres}-$CreS_{K369C}$	This study
YL062	ZG216 + pCT155-P_{cres}-$CreS_{WT}$	This study
**Plasmids**		
pCT155-P_{van}-*3xM2-didA*	pCT155-based parental plasmid for cloning (medium copy number, pJS14-based, $chlor^R$)	[28]
pCT133-P_{van}-*3xM2-didA*	pCT133-based parental plasmid for cloning (low copy number, pMR20-based, tet^R)	[28]
pHis17-CreSΔN27	pHis17-based plasmid for expressing $CreS_{\Delta N27}$ (residues 28-457)	This study
pHis17-CreSN274RtoA	pHis17-based plasmid for expressing $CreS_{N27RtoA}$ (all Arg mutated to Ala in residues 1-27)	This study
pHis17-CreSRod	pHis17-based plasmid for expressing $CreS_{Rod}$ (residues 80-444)	This study

pFE127	pHis17-based plasmid for expressing CreS-His ($CreS_{wt}$ followed by GSHHHHHH)	This study
pHis17-CreSΔ392-412-His	pHis17-based plasmid for expressing $CreS_{\Delta 392\text{-}412}$-His (residues 392-412 deleted)	This study
pHis17-CreSΔ413-433-His	pHis17-based plasmid for expressing $CreS_{\Delta 413\text{-}433}$-His (residues 413-433 deleted)	This study
pHis17-CreS413-433Vim-His	pHis17-based plasmid for expressing $CreS_{413\text{-}433Vim}$-His (residues 413-433 replaced by the homologous region in human vimentin)	This study
pMECS-NB13-HA-His	pMECS-based phagemid for expressing NB13-HA-His, a CreS-specific nanobody followed an HA tag and a 6x His tag	This study (provided by VIB)
pET-22b-MB13-His	pET-22b based plasmid for expressing MB13-His, a CreS-specific megabody with a C-terminal a 6x His tag	This study
pCT133-P_{cres}-$CreS_{wt}$	pCT133 based plasmid for expressing $CreS_{wt}$ under its native promoter	This study
pCT133-P_{cres}-$CreS_{\Delta N27}$	pCT133 based plasmid for expressing $CreS_{\Delta N27}$ (aa 1-27 deleted) under its native promoter	This study
pCT133-P_{cres}-$CreS_{M443C}$	pCT133 based plasmid for expressing $CreS_{M443C}$ under its native promoter	This study
pCT133-P_{cres}-$CreS_{E43C+S74C}$	pCT133 based plasmid for expressing $CreS_{E43C+S74C}$ under its native promoter	This study
pCT133-P_{cres}-$CreS_{E57C+E63C}$	pCT133 based plasmid for expressing $CreS_{E57C+E63C}$ under its native promoter	This study
pCT133-P_{cres}-$CreS_{R419C+S34C}$	pCT133 based plasmid for expressing $CreS_{R419C+S34C}$ under its native promoter	This study
pCT133-P_{cres}-$CreS_{R419C+A37C}$	pCT133 based plasmid for expressing $CreS_{R419C+A37C}$ under its native promoter	This study
pCT133-P_{cres}-$CreS_{K369C+T131C}$	pCT133 based plasmid for expressing $CreS_{K369C+T131C}$ under its native promoter	This study
pCT133-P_{cres}-$CreS_{K369C+A134C}$	pCT133 based plasmid for expressing $CreS_{K369C+A134C}$ under its native promoter	This study
pCT133-P_{cres}-$CreS_{R384C+T120C}$	pCT133 based plasmid for expressing $CreS_{R384C+T120C}$ under its native promoter	This study
pCT133-P_{cres}-$CreS_{K296C+Q204C}$	pCT133 based plasmid for expressing $CreS_{K296C+Q204C}$ under its native promoter	This study
pCT133-P_{cres}-$CreS_{K296C+A207C}$	pCT133 based plasmid for expressing $CreS_{K296C+A207C}$ under its native promoter	This study
pCT133-P_{cres}-$CreS_{A282C+D221C}$	pCT133 based plasmid for expressing $CreS_{A282C+D221C}$ under its native promoter	This study
pCT133-P_{cres}-$CreS_{Q204C}$	pCT133 based plasmid for expressing $CreS_{Q204C}$ under its native promoter	This study
pCT133-P_{cres}-$CreS_{K296C}$	pCT133 based plasmid for expressing $CreS_{K296C}$ under its native promoter	This study
pCT133-P_{cres}-$CreS_{T131C}$	pCT133 based plasmid for expressing $CreS_{T131C}$ under its native promoter	This study
pCT133-P_{cres}-$CreS_{K369C}$	pCT133 based plasmid for expressing $CreS_{K369C}$ under its native promoter	This study
pCT155-P_{cres}-$CreS_{WT}$	pCT155 based plasmid for expressing $CreS_{wt}$ under its native promoter	This study

Table S2. Amino acid sequences of proteins used in this study

CreS *Caulobacter crescentus* crescentin (GenBank accession identifier: ACL97278.1)

MRLLSKNSRETKNGKPTVLGDEARAEAMQHQIESTQAIGQRYETIHGGLDSIGRVMEHLKAIEPLIAEIRG
PVSQEFEARRAEHAELIAVRANLDQAQRQIALIQAEEREVSARLAAAETALGESDARRQTQDAALEDNALE
IDRLRNALLQSDLKVSSLDASLRDATARIEHLVQDVEGLRVQAQDIDARRGDAEAALARANQDNALLGEEA
ATLKKRVDQAGLDLARLSRIETDLEAQLAAERARVQAVENALAAHQADSGRTIRGLESQVEANRAEISALQ
TRLETATGRADKLEEMNGQISARLADSSAQQKAVERRAGDLNVALERALDRIRALEEEADGLRQRHAGVDT
ARATAIERADQLAKSAVAQEKALKRAEERAQQLRARLDAMQEAQDQVRRDHEAKIAELQATIERLTSEAAL
AEGALEAARRDRSRLQMALLGASDGDVAASA

CreS stutter mutant (SAT insertion before 406)

MRLLSKNSRETKNGKPTVLGDEARAEAMQHQIESTQAIGQRYETIHGGLDSIGRVMEHLKAIEPLIAEIRG
PVSQEFEARRAEHAELIAVRANLDQAQRQIALIQAEEREVSARLAAAETALGESDARRQTQDAALEDNALE
IDRLRNALLQSDLKVSSLDASLRDATARIEHLVQDVEGLRVQAQDIDARRGDAEAALARANQDNALLGEEA
ATLKKRVDQAGLDLARLSRIETDLEAQLAAERARVQAVENALAAHQADSGRTIRGLESQVEANRAEISALQ
TRLETATGRADKLEEMNGQISARLADSSAQQKAVERRAGDLNVALERALDRIRALEEEADGLRQRHAGVDT
ARATAIERADQLAKSAVAQEKALKRAEERAQQLRARLDAMQEAQDQVRRDSATHEAKIAELQATIERLTSE
AALAEGALEAARRDRSRLQMALLGASDGDVAASA

Nanobody NB13 (HA-tag, 6×His-tag)

QVQLQESGGGLVQTGGSLRLSCASSRSIDGINIMRWYRQAPGKQRGMVAVVTGWGSTNYVDSVKGRFIISR
DSAKDTVYLQMNNLKPEDTAVYSCNAIYRGSEYWGQGTQVTVSSAAAYPYDVPDYGSHHHHHH

Megabody MB13 (NB13, YgjK, 10×His-tag)

EVQLQESGGGLVYKEETQSGLNNYARVVEKGQYDSLEIPAQVAASWESGRDDAAVFGFIDKEQLDKYVANG
GKRSDWTVKFAENRSQDGTLLGYSLLQESVDQASYMYSDNHYLAEMATILGKPEEAKRYRQLAQQLADYIN
TCMFDPTTQFYYDVRIEDKPLANGCAGKPIVERGKGPEGWSPLFNGAATQANADAVVKVMLDPKEFNTFVP
LGTAALTNPAFGADIYWRGRVWVDQFWFGLKGMERYGYRDDALKLADTFFRHAKGLTADGPIQENYNPLTG
AQQGAPNFSWSAAHLYMLYNDFFRKQASGGGSGGGGSGGGGSGNADNYKNVINRTGAPQYMKDYDYDDHQR
FNPFFDLGAWHGHLLPDGPNTMGGFPGVALLTEEYINFMASNFDRLTVWQDGKKVDFTLEAYSIPGALVQK
LTAKDVQVEMTLRFATPRTSLLETKITSNKPLDLVWDGELLEKLEAKEGKPLSDKTIAGEYPDYQRKISAT
RDGLKVTFGKVRATWDLLTSGESEYQVHKSLPVQTEINGNRFTSKAHINGSTTLYTTYSHLLTAQEVSKEQ
MQIRDILARPAFYLTASQQRWEEYLKKGLTNPDATPEQTRVAVKAIETLNGNWRSPGGAVKFNTVTPSVTG
RWFSGNQTWPWDTWKQAFAMAHFNPDIAKENIRAVFSWQIQPGDSVRPQDVGFVPDLIAWNLSPERGGDGG
NWNERNTKPSLAAWSVMEVYNVTQDKTWVAEMYPKLVAYHDWWLRNRDHNGNGVPEYGATRDKAHNTESGE
MLFTVKKDSLRLSCASSRSIDGINIMRWYRQAPGKQRGMVAVVTGWGSTNYVDSVKGRFIISRDSAKDTVY
LQMNNLKPEDTAVYSCNAIYRGSEYWGQGTQVTVSSGENLYFQGSHHHHHHHHHH

Table S3. Cryo-EM data collection and processing statistics

	**CreS$_{sat}$**				**CreS$_{wt}$**			
	Small box		Large box		Small box		Large box	
	SAT-SB-C2	SAT-SB-C1	SAT-LB-C2	SAT-LB-C1	WT-SB-C2	WT-SB-C1	WT-LB-C2	WT-LB-C1
EMDB ID	EMD-15398	EMD-15395	EMD-15476	EMD-15446	EMD-15402	EMD-15401	EMD-15473	EMD-15465
PDB ID	8AFH	8AFE	8AJB	8AHL	8AFM	8AFL	8AIX	8AIA
**Data collection and processing**								
Microscope	Titan Krios IV (eBIC)				Titan Krios II (MRC LMB)			
Accelerating voltage (kV)	300				300			
Camera	Gatan K3				Falcon IV			
Micrographs (collected/selected)	13330 / 10925				13557 / 9553			
Nominal magnification	81000x				75000x			
Pixel size (Å/pixel)	0.53[a]				1.08			
Dose rate (e^-/pixel/s)	25				4			
Total exposure (e^-/Å^2)	53				34			
Frame rate (ms)	60				4 (EER - electron event representation)			
No. of frames	40				2410			
Defocus (μm)	0.4-2.6				0.4-4.0			
No. of particels (extraction)	869812				1211745			
No. of particels (reconstruction)	599993	1199985	585252	1170504	550575	1101149	550574	1101148
Map resolution (half-map)[b] (Å)	3.85	3.34	4.26	4.11	4.8	4.36	5.78	5.14
Map resolution (model-map)[c] (Å)	4.11	3.79	4.78	4.75	5.27	5.01	7.07	6.34
Pixel size (reconstruction, Å/px)	1.325	1.325	1.59	1.59	1.728	1.728	1.944	1.944
Box size (reconstruction, px)	320	320	600	600	250	250	500	500
Point group symmetry	C2	C1	C2	C1	C2	C1	C2	C1
**Model Statistics**								

Model-map correlation coeffcient[d] - CC_{volume}/CC_{mask}	0.70/0.66	0.70/0.685	0.64/0.55	0.70/0.64	0.64/0.60	0.66/0.63	0.69/0.67	0.71/0.67
No. of atoms								
Protein	13578	6789	30112	15056	7846	3923	21930	10965
R.m.s deviations[e]								
Bond lengths (Å)	0.006	0.006	0.007	0.006	0.005	0.004	0.007	0.007
Bond angles (°)	0.981	0.677	1.251	1.019	0.995	0.723	1.265	1.072
Ramachadran plot[e]								
Favored (%)	95.1	95.1	95.2	95.2	91.6	91.6	89.2	89.2
Allowed (%)	4.9	4.9	4.8	4.8	8.4	8.4	10.8	10.8
Outliers (%)	0.0	0.0	0.0	0.0	0.0	0.0	0.0	0.0
Rotamer outliers[e]	0.00	0.00	0.13	0.13	0.25	0.25	0.09	0.09
Clash score[e]	14.81	13.85	29.63	29.56	17.91	18.17	48.71	48.51
Molprobity score[e]	2.02	1.99	2.29	2.29	2.25	2.26	2.74	2.73
Cβ outliers[e]	0.0	0.0	0.0	0.0	0.0	0.0	0.0	0.0

[a] Super resolution pixel size. The physical pixel size is 1.06 Å/pixel.

[b] Based on FSC between two independently calculated half maps (FSC cutoff 0.143) [9, 10].

[c] Based on model-map FSC between the cryo-EM map and a map calculated based on the atomic model specifying the cryo-EM map resolution (FSC cutoff 0.5) [10].

[d] Atomic coordinates were refined against the cryo-EM map in real space using Phenix [17]. CC_{mask} and CC_{volume} are defined as in [29].

[e] Based on the criteria of MolProbity [20].

Table S4. Comparison of residue-residue contacts between $CreS_{sat}$ and $CreS_{wt}$.

Region	Type	Residue A[a]	Residue B[a]	$CreS_{wt}$[b] (Å)	$CreS_{sat}$[b] (Å)
Middle	Inter-dimer, lateral	K369	T131	8 (9)	8 (9)
Middle	Intra-dimer	K369	K369	17 (15)	16 (14)
Middle	Intra-dimer	T131	T131	16 (15)	15 (14)
Middle	Inter-dimer, lateral	K369	A134	7 (10)	8 (10)
Middle	Inter-dimer, lateral	R384	T120	8 (10)	8 (9)
Crossover	Inter-dimer, lateral	K296	Q204	11 (11)	10 (11)
Crossover	Intra-dimer	K296	K296	N/A[c]	16 (14)
Crossover	Intra-dimer	Q204	Q204	17 (15)	15 (14)
Crossover	Inter-dimer, lateral	K296	A207	8 (10)	7 (9)
Crossover	Inter-dimer, lateral	A282	D221	N/A[c]	6 (6)
NN	Inter-dimer, longitudinal	E43	S74	12 (13)	7 (7)
NN	Inter-dimer, longitudinal	E57	E63	12 (14)	8 (9)
NN	Intra-monomer	E57	E63	11 (10)	12 (11)
CC	Inter-dimer, longitudinal	M443 (446)	M443 (446)	N/A[c]	12 (10)
CC	Intra-dimer	M443 (446)	M443 (446)	18 (16)	18 (17)
NC	Inter-dimer, longitudinal	R419 (422)	S34	N/A[c]	12 (12)
NC	Inter-dimer, longitudinal	R419 (422)	A37	N/A[c]	10 (9)

[a] Based on $CreS_{wt}$ numbering. Values in parentheses represent residue number based on $CreS_{sat}$ numbering.

[b] Cβ-Cβ distance between two residues. Values in parentheses represent the Cα-Cα distance. For a given pair, the distance shown is the shortest among all equivalent pairs related by pseudo symmetry.

[c] At least one of the two residues is absent in the atomic model due to disorder or an ambiguity in model building.

REFERENCES

1. Zheng SQ, Palovcak E, Armache JP*, et al.* (2017) MotionCor2: anisotropic correction of beam-induced motion for improved cryo-electron microscopy. *Nat. Methods* 14(4):331-332.
2. Rohou A & Grigorieff N (2015) CTFFIND4: Fast and accurate defocus estimation from electron micrographs. *J Struct Biol* 192(2):216-221.
3. Bepler T, Kelley K, Noble AJ, & Berger B (2020) Topaz-Denoise: general deep denoising models for cryoEM and cryoET. *Nat Commun* 11(1):5208.
4. Bepler T, Morin A, Rapp M*, et al.* (2019) Positive-unlabeled convolutional neural networks for particle picking in cryo-electron micrographs. *Nat Methods* 16(11):1153-1160.
5. Scheres SH (2012) RELION: implementation of a Bayesian approach to cryo-EM structure determination. *J. Struct. Biol.* 180(3):519-530.
6. Punjani A, Rubinstein JL, Fleet DJ, & Brubaker MA (2017) cryoSPARC: algorithms for rapid unsupervised cryo-EM structure determination. *Nat Methods* 14(3):290-296.
7. Zivanov J, Nakane T & Scheres SHW (2019) A Bayesian approach to beam-induced motion correction in cryo-EM single-particle analysis. *IUCrJ* 6(Pt 1):5-17.
8. Li Y, Zhou M, Hu Q*, et al.* (2017) Mechanistic insights into caspase-9 activation by the structure of the apoptosome holoenzyme. *Proc Natl Acad Sci U S A* 114(7):1542-1547.
9. Scheres SH & Chen S (2012) Prevention of overfitting in cryo-EM structure determination. *Nat. Methods* 9(9):853-854.
10. Rosenthal PB & Henderson R (2003) Optimal determination of particle orientation, absolute hand, and contrast loss in single-particle electron cryomicroscopy. *J. Mol. Biol.* 333(4):721-745.
11. Sanchez-Garcia R, Gomez-Blanco J, Cuervo A*, et al.* (2021) DeepEMhancer: a deep learning solution for cryo-EM volume post-processing. *Commun Biol* 4(1):874.
12. Fang Q, Zhu D, Agarkova I*, et al.* (2019) Near-atomic structure of a giant virus. *Nat Commun* 10(1):388.
13. Guzenko D & Strelkov SV (2018) CCFold: rapid and accurate prediction of coiled-coil structures and application to modelling intermediate filaments. *Bioinformatics* 34(2):215-222.
14. Waterhouse A, Bertoni M, Bienert S*, et al.* (2018) SWISS-MODEL: homology modelling of protein structures and complexes. *Nucleic Acids Res* 46(W1):W296-W303.
15. Pettersen EF, Goddard TD, Huang CC*, et al.* (2004) UCSF Chimera--a visualization system for exploratory research and analysis. *J. Comput. Chem.* 25(13):1605-1612.
16. Emsley P, Lohkamp B, Scott WG, & Cowtan K (2010) Features and development of Coot. *Acta Crystallogr. D Biol. Crystallogr.* 66(Pt 4):486-501.
17. Adams PD, Afonine PV, Bunkoczi G*, et al.* (2010) PHENIX: a comprehensive Python-based system for macromolecular structure solution. *Acta Crystallogr. D Biol. Crystallogr.* 66(Pt 2):213-221.
18. Afonine PV, Poon BK, Read RJ*, et al.* (2018) Real-space refinement in Phenix for cryo-EM and crystallography. *BioRxiv*:249607.
19. Brown JH, Cohen C & Parry DA (1996) Heptad breaks in alpha-helical coiled coils: stutters and stammers. *Proteins* 26(2):134-145.
20. Chen VB, Arendall WB, 3rd, Headd JJ*, et al.* (2010) MolProbity: all-atom structure validation for macromolecular crystallography. *Acta Crystallogr. D Biol. Crystallogr.* 66(Pt 1):12-21.
21. Chernyatina AA, Nicolet S, Aebi U, Herrmann H, & Strelkov SV (2012) Atomic structure of the vimentin central alpha-helical domain and its implications for intermediate filament assembly. *Proc Natl Acad Sci U S A* 109(34):13620-13625.

22. Vermeire PJ, Stalmans G, Lilina AV*, et al.* (2021) Molecular Interactions Driving Intermediate Filament Assembly. *Cells* 10(9).
23. Cabeen MT, Herrmann H & Jacobs-Wagner C (2011) The domain organization of the bacterial intermediate filament-like protein crescentin is important for assembly and function. *Cytoskeleton (Hoboken)* 68(4):205-219.
24. Evinger M & Agabian N (1977) Envelope-associated nucleoid from Caulobacter crescentus stalked and swarmer cells. *J Bacteriol* 132(1):294-301.
25. Gitai Z, Dye N & Shapiro L (2004) An actin-like gene can determine cell polarity in bacteria. *Proc Natl Acad Sci U S A* 101(23):8643-8648.
26. Ingerson-Mahar M, Briegel A, Werner JN, Jensen GJ, & Gitai Z (2010) The metabolic enzyme CTP synthase forms cytoskeletal filaments. *Nat Cell Biol* 12(8):739-746.
27. Cabeen MT, Charbon G, Vollmer W*, et al.* (2009) Bacterial cell curvature through mechanical control of cell growth. *EMBO J* 28(9):1208-1219.
28. Modell JW, Kambara TK, Perchuk BS, & Laub MT (2014) A DNA damage-induced, SOS-independent checkpoint regulates cell division in *Caulobacter crescentus*. *PLoS Biol* 12(10):e1001977.
29. Afonine PV, Klaholz BP, Moriarty NW*, et al.* (2018) New tools for the analysis and validation of cryo-EM maps and atomic models. *Acta Crystallogr D Struct Biol* 74(Pt 9):814-840.
